# A versatile reactive layer toward ultra-long lifespan lithium metal anodes

**DOI:** 10.1093/nsr/nwae421

**Published:** 2024-12-03

**Authors:** Jinlun Wu, Yuheng Lu, Xianlan Ke, Linying Zheng, Rongfeng Liao, Dingcai Wu

**Affiliations:** PCFM Lab and GDHPRC Lab, School of Chemistry, Sun Yat-sen University, Guangzhou 510006, China; PCFM Lab and GDHPRC Lab, School of Chemistry, Sun Yat-sen University, Guangzhou 510006, China; PCFM Lab and GDHPRC Lab, School of Chemistry, Sun Yat-sen University, Guangzhou 510006, China; PCFM Lab and GDHPRC Lab, School of Chemistry, Sun Yat-sen University, Guangzhou 510006, China; PCFM Lab and GDHPRC Lab, School of Chemistry, Sun Yat-sen University, Guangzhou 510006, China; PCFM Lab and GDHPRC Lab, School of Chemistry, Sun Yat-sen University, Guangzhou 510006, China

**Keywords:** versatile reactive layers, robust anode/electrolyte interfaces, ultralong-term cycling, lithium metal anodes

## Abstract

Unstable anode/electrolyte interfaces have significantly hindered the development of lithium (Li) metal batteries under high rates and large capacities. In this study, a versatile reactive layer based on sulfur-selenium crosslinked polyacrylonitrile brushes has been developed by a combined strategy of polymer topology design and chemical crosslinking. The sulfur-selenium crosslinked polyacrylonitrile side-chains can react with Li to generate passivated Li_2_S-Li_2_Se-containing solid electrolyte interphase while 3D lithiophilic porous nanonetworks enable Li penetration, contributing to achieving rapid and uniform Li ion flux and a dendrite-free anode. With these merits, ultralong-term stable cycling (over 1 year and 4 months) at a high current density of 10 mA cm^−2^ has been achieved for the protected Li anodes. Moreover, even when tested in high-loading Li|NCM622 cell (21.6 mg cm^−2^) and Li-S cell with a low negative to positive electrode capacity ratio (1.4), stable cycling performances can also be achieved.

## Teaser

Sulfur-selenium crosslinked polyacrylonitrile brushes are developed as versatile reactive layers to construct robust anode/electrolyte interfaces toward ultrastable lithium metal anodes.

## INTRODUCTION

Lithium (Li) metal is widely considered as one of the most promising alternative anode materials due to its lower electrochemical potential (−3.04 V) relative to the standard hydrogen electrode and ultrahigh theoretical capacity (∼3860 mAh g^−1^) in comparison with the classical graphite anode (∼372 mAh g^−1^) [1]. Motivated by the flourish of Li metal anodes, the utilization of cathode materials has been extended from Li-rich intercalated cathodes (e.g. LiFePO_4_, LiCoO_2_, and LiNi_x_Co_y_Mn_1−x−y_O_2_) to Li-free conversion cathodes (e.g. sulfur and air) [2]. Nevertheless, lithium metal batteries (LMBs) are largely plagued by unstable anode/electrolyte interfaces and the growth of dendrites [3]. In the actual situation, Li anodes with low reduction potential are susceptible to reacting with electrolyte salts/solvents to form passivation layers, commonly termed as solid electrolyte interphase (SEI) [4]. Such a native SEI is usually discontinuous and nonuniform so that a fresh Li surface can be easily exposed to seriously consume the electrolyte, giving rise to constant capacity decay and insufficient cycle lifespan [5]. Furthermore, local Li ion enrichment caused by the uneven surface of Li anode would lead to non-planar Li deposition in the form of mossy or dendritic structures, which could penetrate the separator to cause an internal short circuit and even catastrophic combustion and explosion [5,6]. What's worse, most LMBs cannot work at high current densities (normally <10 mA cm^−2^) and high areal capacities (normally <5 mAh cm^−2^), and their negative to positive electrode capacity (N/P) ratios are relatively high (normally >10), greatly restricting their practical applications. Therefore, it is of vital significance to develop highly stable Li anodes for improving the safety and cycle life of LMBs.

**Figure 1.**
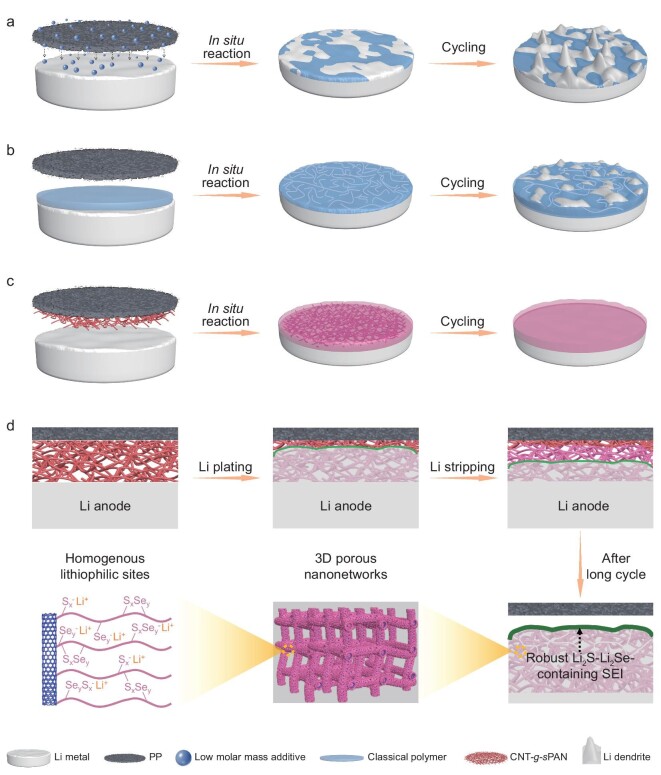
Schematic illustration of *in situ* formed anode/electrolyte interfaces and corresponding Li deposition behaviors. (a) Continuous irreversible consumption of low molar mass additives in electrolytes would lead to unstable electrochemical processes during long-term cycling, followed by formation of unstable SEI overwhelmingly comprised of Li salts with insufficient flexibility, giving rise to continuous defects and cracks as well as non-uniform Li ion flux. (b) Classical polymers with linear and nonporous structure characteristics can hardly provide sufficient spaces and lithiophilic groups to alleviate volume changes and achieve rapid and uniform Li ion flux, and their low mechanical robustness cannot effectively suppress dendrite growth during Li plating/stripping under harsh performance evaluation conditions. (c) Schematic illustration of using CNT-*g*-*s*PAN reactive layer to construct a robust Li anode. (d) For the reactive CNT-*g*-*s*PAN, its sulfur-selenium crosslinked PAN side-chains are able to react with Li to generate robust Li_2_S-Li_2_Se-containing SEI during cycling, contributing to a stable anode/electrolyte interface with rapid and uniform Li ion flux; after a period of charging/discharging, the lithiophilic CNT-*g*-*s*PAN would be embedded in the Li anode to provide 3D porous nanonetworks for Li plating/stripping and inhibition of Li dendrite growth.

Among the efforts to improve the stability of Li anodes, construction or modification of SEI could be the most crucial, because such an anode/electrolyte interface is responsible for Li ion transport and mechanical accommodation of repeated Li plating/stripping. Various functional electrolyte additives (e.g. fluoroethylene carbonate, LiNO_3_ and organothiol) have been adopted to modify surface morphologies and physicochemical properties of SEI. The developed SEI generated by these compounds has been proved to involve specific compositions (such as LiF, Li_3_N and Li_2_S) with highly ionic conductivity [7–9]. Generally, continuous irreversible consumption of low molar mass additives in electrolytes would lead to unstable electrochemical processes during long-term cycling and form unstable SEI overwhelmingly comprised of Li salts with insufficient flexibility, giving rise to continuous defects and cracks as well as non-uniform Li ion flux (Fig. 1a) [10,11]. Polymers might offer a good alternative to achieve reliable SEI due to their relatively high stability, superior flexibility and excellent structural tunability. However, due to their linear and nonporous structure characteristics, classical polymers can hardly provide sufficient spaces and lithiophilic groups to alleviate volume changes and achieve rapid and uniform Li ion flux. Furthermore, their low mechanical robustness cannot effectively suppress dendrite growth during Li plating/stripping under harsh performance evaluation conditions, such as high current density, large areal capacity and limited Li supply (Fig. 1b). Therefore, from the perspective of material engineering, the fabrication of polymers with porous structures, rigid skeletons and homogenous lithiophilic sites is highly desirable yet remains elusive to realize ultrastable Li metal anodes at high current densities and large areal capacities.

Herein, a new class of sulfur-selenium crosslinked polyacrylonitrile brush (CNT-*g*-*s*PAN) was synthesized based on the union of polymer topology design and chemical crosslinking strategy and utilized as a versatile reactive layer to ameliorate the anode/electrolyte interface (Fig. 1c, d). During repeated cycles, the sulfur-selenium crosslinked polyacrylonitrile side-chains in CNT-*g*-*s*PAN are able to react with Li to form passivated Li_2_S-Li_2_Se-containing SEI, while the 3D lithiophilic porous nanonetworks with conductive carbon nanotube substrates enable Li penetration into pores. The passivated Li_2_S-Li_2_Se–containing SEI is able to promote rapid and uniform Li ion flux. The 3D porous nanonetworks can provide accessible spaces for stable Li plating/stripping and effectively reduce local current density. As a result, ultralong-term stable cycling for over 12 000 h (>1 year and 4 months) at a high current density of 10 mA cm^−2^ and transcendent reversible Li plating/stripping for over 3600 h at a large areal capacity of 21 mAh cm^−2^ have been achieved for Li metal anodes. When paired with high-loading LiNi_0.6_Co_0.2_Mn_0.2_O_2_ (NCM622) cathode (21.6 mg cm^−2^), an excellent capacity retention of 94% can be retained after 100 cycles at 1 C. Moreover, at a low N/P ratio (1.4), the lithium-sulfur (Li-S) cell still delivers an average areal capacity of 3.4 mAh cm^−2^ after 200 cycles.

## RESULTS AND DISCUSSION

### Design and structure of CNT-*g*-*s*PAN

**Figure 2.**
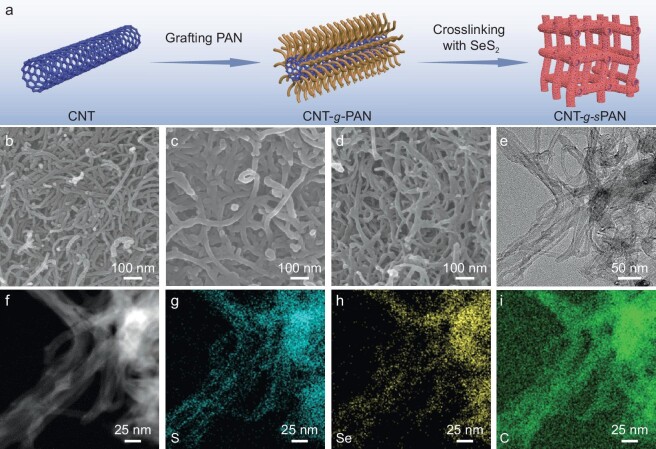
Morphological characterizations of CNT-*g*-*s*PAN. (a) Synthetic scheme of CNT-*g*-*s*PAN. SEM images of (b) CNT, (c) CNT-*g*-PAN and (d) CNT-*g*-*s*PAN. (e–i) TEM images and elemental mapping analysis of CNT-*g*-*s*PAN.

The synthetic scheme of CNT-*g*-*s*PAN is illustrated in Fig. 2a. Polyacrylonitrile (PAN) side-chains were first grafted onto the modified surfaces of carbon nanotubes (CNTs) via a free radical polymerization strategy to prepare CNT-*g*-PAN brushes. Scanning electron microscopy (SEM) images reveal that CNT-*g*-PAN inherits a typical nanomorphology of CNT, with an increase in average diameter, suggesting successful growth of PAN side-chains (Fig. 2b, c). The mass content of PAN was determined by thermogravimetric analysis (TGA), which was calculated to be 39.2 wt% from the curves of CNT modified by vinyl group (CNT-C=C), PAN and CNT-*g*-PAN (Fig. S1). Subsequently, crosslinking reaction between CNT-*g*-PAN and SeS_2_ was carried out in an alumina crucible wrapped with aluminum foil at 380°C for 8 h under N_2_ flow, and the mixture was then subjected to heat treatment at 300°C for another 4 h in an opened alumina crucible under N_2_ flow to remove any SeS_2_ surplus, leading to formation of CNT-*g*-*s*PAN. As shown in Fig. 2d, the as-obtained CNT-*g*-*s*PAN still retains 1D nanotube morphology without any obvious SeS_2_ aggregation. Transmission electron microscope (TEM) images and corresponding elemental mapping indicate homogeneous distribution of S and Se in CNT-*g*-*s*PAN (Fig. 2e–i). In sharp contrast, a control sample (i.e. CNT/*s*PAN) prepared by crosslinking reaction of a mixture of CNT, PAN and SeS_2_ shows ill-defined and quasi-nonporous structures with large agglomerations (Fig. S2). These results validate that the unique nanoarchitecture of CNT-*g*-PAN brushes is the prerequisite for the successful construction of well-defined CNT-*g*-*s*PAN.

**Figure 3.**
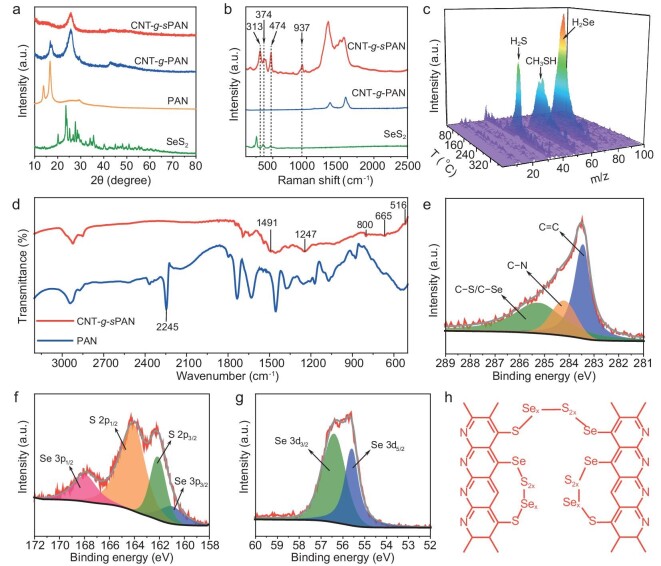
Investigation of chemical compositions of CNT-*g*-*s*PAN. (a) XRD patterns of SeS_2_, PAN, CNT-*g*-PAN and CNT-*g*-*s*PAN. (b) Raman spectra of SeS_2_, CNT-*g*-PAN and CNT-*g*-*s*PAN. (c) *In situ* time-resolved mass spectra obtained during the crosslinking of CNT-*g*-PAN with SeS_2_. (d) FT-IR spectra of PAN and CNT-*g*-*s*PAN. (e) C 1s, (f) S 2p/Se 3p and (g) Se 3d high-resolution XPS spectra of CNT-*g*-*s*PAN. (h) A possible chemical structure of crosslinked PAN side-chains in CNT-*g*-*s*PAN.

After the crosslinking reaction, the as-obtained CNT-*g*-*s*PAN doesn't show any characteristic peaks of PAN and SeS_2_, according to the X-ray diffraction (XRD) patterns (Fig. 3a) [12]. As shown in the Raman spectra of Fig. 3b, the peaks of CNT-*g*-*s*PAN located at 313 and 374 cm^−1^ result from its C–S and S–Se bonds, respectively, and the peaks at 474 and 937 cm^−1^ originate from its S–S bond [13]. These results suggest that SeS_2_ and CNT-*g*-PAN undergo a crosslinking reaction to form new covalent bonds. According to TGA curves of CNT-*g*-*s*PAN, CNT-C=C, and sulfur-selenium crosslinked PAN (*s*PAN), the mass content of *s*PAN in CNT-*g*-*s*PAN is calculated to be 71.1 wt% (Fig. S1). TGA coupled with mass spectroscopy was conducted to monitor the crosslinking reaction process between CNT-*g*-PAN and SeS_2_. As shown in Fig. 3c, H_2_S (m/z = 34), CH_3_SH (m/z = 48) and H_2_Se (m/z = 81) are detected, suggesting an obvious dehydrogenation reaction between PAN and SeS_2_. This dehydrogenation process enables the cyclization and crosslinking of PAN side-chains, which could be supported by Fourier transform infrared (FT-IR) and X-ray photoelectron spectroscopy (XPS) analyses. In the FT-IR spectra of Fig. 3d, the characteristic peak at 2245 cm^−1^ for C≡N of PAN side-chains basically vanished in CNT-*g*-*s*PAN [12]. For CNT-*g*-*s*PAN, the peaks at 516, 665, 1247 and 1491 cm^−1^ originate from S–S, C–S, C=N and C=C bonds, respectively, and the ring breathing of cyclic structure appears at 800 cm^−1^ [13]. As illustrated in Fig. 3e, C 1s high-resolution XPS spectrum confirms the covalent bonds between S/Se and C. In S 2p spectrum, S–Se (164.1 eV) and S–S (165.2 eV) bonds are observed, while the peaks at 167.9 and 162.1 eV are attributed to Se 3p_1/2_ and Se 3p_3/2_ (Fig. 3f) [14,15]. In addition, the peaks at 56.4 and 55.5 eV in Se 3d spectrum validate the existence of Se–S bond (Fig. 3g) [16]. According to the aforementioned analyses, PAN side-chains undergo consecutive dehydrogenation reactions to generate N-heterocyclic structures with Se_x_S_y_ crosslinking bridges, and a possible chemical structure of sulfur-selenium crosslinked PAN side-chains in CNT-*g*-*s*PAN is proposed in Fig. 3h.

CNT-*g*-*s*PAN was blade-coated as a reactive layer onto one side of Celgard 2325 porous polyolefin (PP) separator. The as-obtained CNT-*g*-*s*PAN@PP separator exhibits good mechanical properties so that it can be folded several times without any detachment of CNT-*g*-*s*PAN (Fig. S3), which is beneficial for adapting to changes in cell assembling and testing. The cross-section SEM image reveals that CNT-*g*-*s*PAN layer with a thickness of ∼12 μm closely adheres to the PP surface (Fig. S4). As shown in Figs S5 and S6, both PP and CNT-*g*-*s*PAN@PP separators possess well-developed porous structures, and their contact angle measurements indicate a better electrolyte affinity of CNT-*g*-*s*PAN@PP than PP, which is beneficial for rapid Li ion transportation and reduced interface impedance.

### Li ion transport and Li plating/stripping behaviors

**Figure 4.**
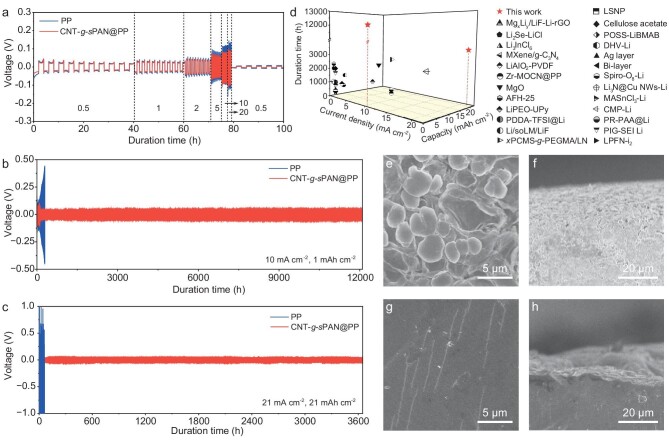
Electrochemical performances of Li|Li symmetric cells. (a) Voltage profiles of Li|Li symmetric cells with CNT-*g*-*s*PAN@PP and PP separators at various current densities in a fixed capacity of 1 mAh cm^−2^. Cycle stability of Li|Li symmetric cells with CNT-*g*-*s*PAN@PP and PP separators at (b) 10 mA cm^−2^ and 1 mAh cm^−2^, as well as (c) 21 mA cm^−2^ and 21 mAh cm^−2^. (d) Comparison of cycle life of Li|Li symmetric cells with CNT-*g*-*s*PAN@PP separators with those with the state-of-the-art Li anodes stabilized by various methods. (e and g) Top-view and (f and h) cross-section SEM images of Li anodes after the test of Li|Li symmetric cells with (e and f) PP and (g and h) CNT-*g*-*s*PAN@PP separators for 100 cycles at 10 mA cm^−2^ and 1 mAh cm^−2^.

According to Fig. S7, the Li ion conductivity of CNT-*g*-*s*PAN@PP is determined as 4.3 × 10^−4^ S cm^−1^, which is much higher than that of the PP separator (1.2 × 10^−4^ S cm^−1^). The Li ion transference number (*t*_Li+_) of CNT-*g*-*s*PAN@PP is also higher than that of PP (0.51 *vs* 0.35), suggesting the CNT-*g*-*s*PAN layer enables kinetically efficient ion diffusion and migration (Fig. S8). Therefore, CNT-*g*-*s*PAN@PP separators show good advantages in reducing the polarization effect and accelerating the Li ion diffusion. The linear sweep voltammetry (LSV) test suggests that CNT-*g*-*s*PAN@PP separator is stable up to 4.9 V (Fig. S9), indicating its high electrochemical stability to pair with sulfur and NCM622 cathodes. Li|Cu cells were assembled to investigate the overpotential and coulombic efficiency (CE) of Li plating/stripping. As shown in Fig. S10, the average CE of CNT-*g*-*s*PAN@PP is 99.7% after 200 cycles at 1 mA cm^−2^ and 1 mAh cm^−2^, indicating that CNT-*g*-*s*PAN can reduce the continuous rupture/reconstruction of SEI and the side reactions between electrolyte and Li. Such a high average CE is favorably comparable to those of previously reported Li|Cu cells with Li metal anodes stabilized by different strategies (Fig. S11). Li|Li symmetric cells were fabricated to understand the reaction kinetics of Li anodes. Cyclic voltammetry (CV) curves show significantly increased current responses in the presence of CNT-*g*-*s*PAN (Fig. S12), suggesting rapid charge transfer and reversible reaction kinetics. Tafel plot of CNT-*g*-*s*PAN@PP exhibits a substantially higher exchange current density (1.71 mA cm^−2^) than that of bare PP (0.02 mA cm^−2^), further demonstrating the faster charge transfer kinetics (Fig. S13) [17]. After an 8-day rest at open-circuit potential, charge-transfer resistance (*R*_ct_) of the cell with CNT-*g*-*s*PAN@PP increases from 27 to 73 Ω whereas the control cell shows greatly enlarged *R*_ct_ from 120 to 650 Ω (Fig. S14), which could be ascribed to the parasitic reaction between Li anode and electrolyte and the generation of a nonuniform SEI layer. Galvanostatic tests were conducted to evaluate the suppressive effect of Li dendrites. When the cycling capacity is fixed at 1 mAh cm^−2^, the cell with CNT-*g*-*s*PAN@PP shows low overpotentials of 15 and 35 mV at 0.5 and 2 mA cm^−2^, respectively, and a small overpotential of only 79 mV can still be obtained even at 20 mA cm^−2^ (Fig. 4a and Fig. S15). An ultralong-term stable Li plating/stripping behavior for over 12 000 h (>1 year and 4 months) is achieved in the cell with CNT-*g*-*s*PAN@PP at a high current density of 10 mA cm^−2^, whereas severe voltage fluctuation is observed in the cell with bare PP (Fig. 4b). As shown in Fig. 4c, even at an ultrahigh current density of 21 mA cm^−2^ and an ultrahigh areal capacity of 21 mAh cm^−2^, the cell with CNT-*g*-*s*PAN@PP still realizes a superior cycle stability for more than 3600 h (>5 months). In sharp contrast, severe voltage fluctuation and rapid cell failure are observed in the cell with bare PP. To the best of our knowledge, such excellent cycle capabilities surpass most of the state-of-the-art Li metal anodes stabilized by various strategies (Fig. 4d), such as artificial SEI layers (e.g. 400 h under a capacity of 1 mAh cm^−2^ at 1 mA cm^−2^) [18–37], electrolyte additives (e.g. 300 h under a capacity of 1 mAh cm^−2^ at 1 mA cm^−2^) [38,39], composite membranes (e.g. 450 h under a capacity of 10 mAh cm^−2^ at 10 mA cm^−2^) [40] and others [41,42].

### Role of CNT-*g*-*s*PAN in stabilizing the Li anode

After the cycling tests, Li|Li symmetric cells were disassembled to further investigate the nanomorphologies and chemical compositions of Li anodes. The top-view and cross-section images show obviously mossy dendrites and accumulated dead Li with porous structure in the cell with PP, which results from the nonuniform Li deposition (Fig. 4e, f). As for the cell with CNT-*g*-*s*PAN@PP separator, a flat and dense structure of the Li anode without obvious dendrites is observed (Fig. 4g, h). Cross-section SEM image and corresponding elemental mapping analysis display relatively homogenous distributions of Li, C, S and Se elements in the Li anode (Fig. S16). The reaction between CNT-*g-s*PAN layer and Li anode was validated in different cycles of Li|Li symmetric cell at 1 mAh cm^−2^ and 10 mA cm^−2^. As shown in Fig. S17, the gradually diminished CNT-*g-s*PAN reactive layer of CNT-*g****-****s*PAN@PP separator indicates that CNT-*g*-*s*PAN reactive layer is gradually embedded in the Li anode during the charge/discharge process. After 100 cycles, CNT-*g*-*s*PAN reactive layer in contact with the Li anode had totally vanished and the corresponding Li anode was further etched to investigate its residuals. The discovery of 3D porous nanonetwork materials confirms that Li is able to penetrate into the pores of CNT-*g****-****s*PAN reactive layer (Fig. S18). The above results demonstrate that the structurally homogeneous and porous CNT-*g*-*s*PAN reactive layer works efficiently to suppress the growth of Li dendrites and provide accessible spaces during Li plating/stripping.

**Figure 5.**
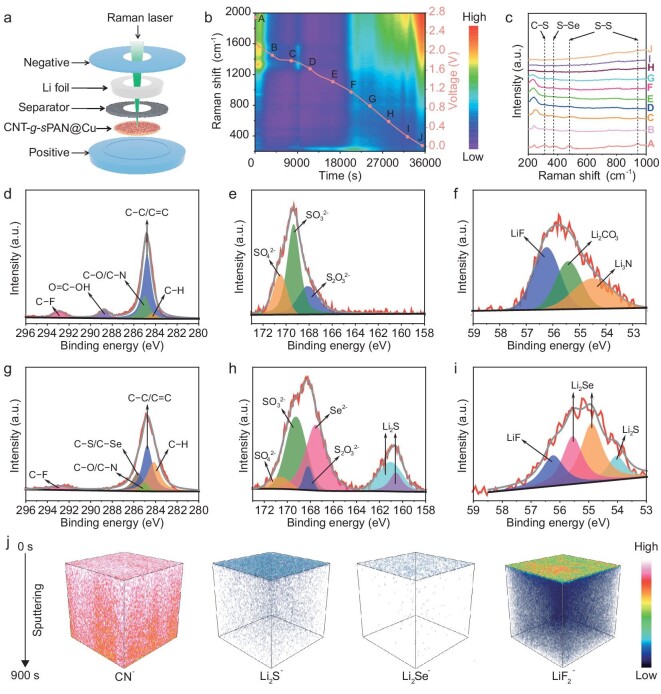
Clarification of the mechanism of CNT*-g*-*s*PAN in stabilizing Li anode. (a) Schematic illustration of the optical cell design that allows for operando optical microscope observation. (b) *In situ* time-resolved Raman spectra obtained during the discharge process of the cell with CNT-*g*-*s*PAN@Cu as working electrode and Li foil as counter and reference electrode, and (c) the selected Raman spectra at different stages. (d) C 1s, (e) S 2p and (f) Li 1s high-resolution XPS spectra of Li anode surface after the test of Li|Li symmetric cell with PP separator for 100 cycles at 10 mA cm^−2^ and 1 mAh cm^−2^. (g) C 1s, (h) S 2p/Se 3p and (i) Li 1s/Se 3d high-resolution XPS spectra of Li anode surface after the test of Li|Li symmetric cell with CNT-*g*-*s*PAN@PP separator for 100 cycles at 10 mA cm^−2^ and 1 mAh cm^−2^. (j) TOF-SIMS based 3D rendering models of CN^−^, Li_2_S^−^, Li_2_Se^−^ and LiF_2_^−^ of the Li anode after the test of Li|Li symmetric cell with CNT-*g*-*s*PAN@PP separator for 100 cycles at 10 mA cm^−2^ and 1 mAh cm^−2^.

The chemical evolution of CNT-*g*-*s*PAN reactive layer was investigated by *in situ* Raman spectroscopy to elucidate the underlying mechanism of Li deposition. The measurement was taken in a home-built electrochemical cell with CNT-*g*-*s*PAN@Cu as the working electrode and Li foil as the counter and reference electrode (Fig. 5a). As illustrated in Fig. 5b, c, Raman signals associated with C–S, S–Se and S–S bonds, which are observed within the range of 300 to 950 cm^−1^ at an open-circuit voltage of 2.7 V [13], gradually decrease during the discharge process, and no discernible characteristic peaks of CNT-*g*-*s*PAN are monitored at 0.01 V, suggesting that CNT-*g*-*s*PAN can react with Li to achieve the construction of SEI. XPS and time-of-flight secondary ion mass spectrometry (TOF-SIMS) were used to investigate the *in situ* generated SEI. In the Li|Li symmetric cell with PP, characteristic peaks of C–F, O=C–OH, C–N/C–O, C–C/C=C and C–H are observed in the C 1 s spectrum (Fig. 5d) [43]. The high-resolution S 2p spectrum can be fitted by three peaks, which are attributed to SO_4_^2−^ (170.5 eV), SO_3_^2−^ (169.3 eV) and S_2_O_3_^2−^ (168.1 eV) (Fig. 5e). Classical SEI components such as LiF (56.2 eV), Li_2_CO_3_ (55.4 eV) and Li_3_N (54.5 eV) are detected in Li 1s spectrum (Fig. 5f) [43]. In sharp contrast, some newly emerged peaks are detected in the Li|Li symmetric cell with CNT-*g*-*s*PAN@PP, including C–S/C–Se (285.6 eV) in C 1s spectrum (Fig. 5g) [12], Li_2_S (160.6 and 161.1 eV) in S 2p spectrum (Fig. 5h) [10], as well as Li_2_S (54.0 eV) and Li_2_Se (55.6 and 54.9 eV) in Li 1s/Se 3d spectrum (Fig. 5i) [44], suggesting that CNT-*g*-*s*PAN is involved in the construction of Li_2_S-Li_2_Se–containing SEI. TOF-SIMS was used to further investigate the 3D distribution of components in the SEI. Fig. S19 and Fig. 5j demonstrate the depth profiles and 3D rendering models of CN^−^, Li_2_S^−^, Li_2_Se^−^ and LiF_2_^−^ secondary-ion species, which could be derived from CNT-*g*-*s*PAN, Li_2_S, Li_2_Se and LiF, respectively [45,46]. The uniform spatial distribution of CN^−^ fragments suggests that CNT-*g*-*s*PAN is embedded in the Li anode after a period of charging/discharging. The content of LiF_2_^−^ fragments is maintained at a relatively high level with increasing sputtering depth. Remarkably, Li_2_S^−^ and Li_2_Se^−^ fragments are predominantly detected in the top surface of SEI, indicating the superiority of CNT-*g*-*s*PAN in constructing the unique Li_2_S-Li_2_Se–containing SEI. The high ionic conductivity of Li_2_S and Li_2_Se enables the rapid and homogeneous transfer of Li ions across the entire electrode surface, thereby preventing the growth of Li dendrites [44].

### Full cell evaluations

**Figure 6.**
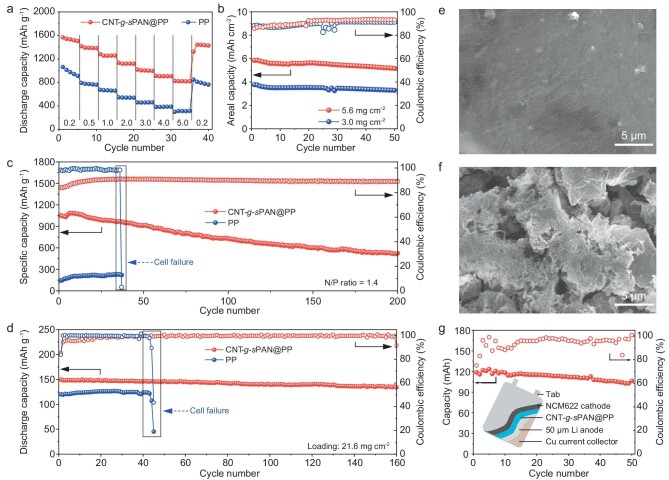
Electrochemical performances of full cells. (a) Rate performance of Li-S cells with PP and CNT-*g*-*s*PAN@PP separators under a sulfur loading of 1 mg cm^−2^. (b) Cycling performance of Li-S cells with CNT-*g*-*s*PAN@PP separators under elevated sulfur loadings. (c) Cycling performance of Li-S cells with PP and CNT-*g*-*s*PAN@PP separators, which couple high sulfur loading cathodes (4.5 mg cm^−2^) with ultrathin Li anodes (50 μm). (d) Cycling performance of Li|NCM622 coin cells with PP and CNT-*g*-*s*PAN@PP separators at charging/discharging of 0.2/1 C. Top-view SEM images of Li anodes after the test of Li|NCM622 coin cells with (e) CNT-*g*-*s*PAN@PP and (f) PP separators for 20 cycles at charging/discharging of 0.2/1 C. (g) Cycling performance of Li|NCM622 pouch cell with CNT-*g*-*s*PAN@PP separator at 0.1 C (inset: schematic diagram of the pouch cell).

The superiority of CNT-*g*-*s*PAN protected Li anodes was demonstrated in full cells with sulfur and NCM622 cathodes. As shown in Fig. S20, the *R*_ct_ of Li-S cells with CNT-*g*-*s*PAN@PP is much lower than that of Li-S cells with PP, indicating that the generation of Li_2_S-Li_2_Se–containing SEI facilitates ion transport. The Li-S cell with CNT-*g*-*s*PAN@PP delivers average discharge capacities of 1533, 1262 and 1123 mAh g^−1^ at 0.2, 1 and 2 C, respectively (Fig. 6a). Even at a high current density of 5 C, a superior discharge capacity of 822 mAh g^−1^ is achieved. When the current density returns to 0.2 C, a high reversible capacity of 1416 mAh g^−1^ can still be retained, demonstrating a robust rate capability. In sharp contrast, the cell with PP displays much inferior discharge capacities at various current densities. Considering that sulfur loading and Li supply are pivotal parameters in high-energy–density Li-S cells, the cells with elevated sulfur loading cathodes and ultrathin Li anodes were assembled. As shown in Fig. 6b and Fig. S21, Li-S cells with sulfur loadings of 3.0 and 5.6 mg cm^−2^ deliver average areal capacities of 3.5 and 5.5 mAh cm^−2^, respectively. Remarkably, even coupling an ultrathin Li anode (50 μm thickness) with a high-loading sulfur cathode of 4.5 mg cm^−2^ (N/P ratio is 1.4), the Li-S full cell still exhibits typical charge-discharge profiles with satisfactory potential plateaus and delivers a high average areal capacity of 3.4 mAh cm^−2^ after 200 cycles at 0.1 C (Fig. 6c and Fig. S22), which is comparable to recently reported Li-S cells (Table S1). In sharp contrast, the cell with PP shows a greatly inferior discharge capacity and fails after only 36 cycles (Fig. 6c), highlighting the significance of CNT-*g*-*s*PAN in construction of ultrastable Li anodes. This can be further supported by the outstanding performance of the cell with high-loading NCM622 cathode (21.6 mg cm^−2^) and lean electrolyte (40 μL). Benefiting from efficient ion transport kinetics with reduced interfacial charge transfer resistance (Fig. S23), the cell with CNT-*g*-*s*PAN@PP demonstrates excellent cycle stability with 94% retention of the initial capacity (150 mAh g^−1^) after 100 cycles at 1 C, much better than that of the cell with bare PP (Fig. 6d and Fig. S24). The Li|NCM622 cell was disassembled to etch the Li anode. The CNT-*g-s*PAN-like 3D porous nanonetworks are observed in the as-obtained residuals, demonstrating that the 3D lithiophilic porous nanonetworks enable Li penetration into pores (Fig. S25). This result suggests that the CNT-*g*-*s*PAN reactive layer can provide accessible spaces to achieve stable Li plating/stripping. Furthermore, the surface morphologies of Li metal anodes after 20 cycles were monitored. As shown in top-view SEM images, the cell with CNT-*g*-*s*PAN@PP shows compact and dendrite-free Li depositions, while highly porous Li dendrites are identified in the cell with PP (Fig. 6e, f). To further demonstrate the potential for actual commercialization, a single-layer 3.6 × 5.6 cm^2^ pouch cell was obtained by assembling the 50 μm Li anode, NCM622 cathode and CNT-*g*-*s*PAN@PP separator (Fig. 6g). The Li|NCM622 pouch cell with a low N/P ratio (1.8) delivers a superior capacity retention of 89% over 50 cycles at 0.1 C, demonstrating the tremendous potential of stable LMBs in practical applications.

## CONCLUSION

We have successfully designed and fabricated a novel class of CNT-*g*-*s*PAN as the versatile reactive layer to achieve the construction of a robust anode/electrolyte interface. The sulfur-selenium crosslinked polyacrylonitrile side-chains are able to react with Li to generate Li_2_S-Li_2_Se–containing SEI. The passivated Li_2_S-Li_2_Se–containing SEI can promote rapid and uniform Li ion flux, while the 3D porous nanonetworks can offer accessible spaces for stable Li plating/stripping and effectively reduce the local current density. With these advantages, the CNT-*g*-*s*PAN@PP protected Li anode can deliver ultralong-term stable cycle life over 12 000 h (>1 year and 4 months) at a high current density of 10 mA cm^−2^ and reversible Li plating/stripping over 3600 h at a large areal capacity of 21 mAh cm^−2^. The Li-S cell with a low N/P ratio (1.4) demonstrates a high average areal capacity of 3.4 mAh cm^−2^ after 200 cycles at 0.1 C. Moreover, a superior initial discharge capacity of 150 mAh g^−1^ and an excellent capacity retention of 94% can still be achieved in the high-loading Li|NCM622 cell (21.6 mg cm^−2^) after 100 cycles at 1 C. We have developed a versatile reactive layer by the union of polymer topology design and chemical crosslinking strategy to optimize the interfacial properties of Li anodes, which is expected to shed some light on the development of ultralong-term and high-energy–density LMBs.

## Supplementary Material

nwae421_Supplemental_File

## References

[1] Lin D, Liu Y, Cui Y et al. Reviving the lithium metal anode for high-energy batteries. Nat Nanotechnol 2017; 12: 194–206. 10.1038/nnano.2017.16 28265117

[2] Zuo W, Luo M, Liu X et al. Li-rich cathodes for rechargeable Li-based batteries: reaction mechanisms and advanced characterization techniques. Energy Environ Sci 2020; 13: 4450–97. 10.1039/D0EE01694B

[3] Sun B, Zhang Q, Xu W et al. A gradient topology host for a dendrite-free lithium metal anode. Nano Energy 2022; 94: 106937. 10.1016/j.nanoen.2022.106937

[4] He X, Bresser D, Passerini S et al. The passivity of lithium electrodes in liquid electrolytes for secondary batteries. Nat Rev Mater 2021; 6: 1036–52. 10.1038/s41578-021-00345-5

[5] Zhao Q, Stalin S, Archer L et al. Stabilizing metal battery anodes through the design of solid electrolyte interphases. Joule 2021; 5: 1119–42. 10.1016/j.joule.2021.03.024

[6] Chen X, Zhao B, Yan C et al. Review on Li deposition in working batteries: from nucleation to early growth. Adv Mater 2021; 33: 2004128. 10.1002/adma.202004128 33432664

[7] Liu Y, Tao X, Wang Y et al. Self-assembled monolayers direct a LiF-rich interphase toward long-life lithium metal batteries. Science 2022; 375: 739–45. 10.1126/science.abn1818 35175797

[8] Tan J, Ye M, Shen J et al. Deciphering the role of LiNO_3_ additives in Li-S batteries. Mater Horiz 2022; 9: 2325–34. 10.1039/D2MH00469K 35766933

[9] Guo W, Zhang W, Si Y et al. Artificial dual solid-electrolyte interfaces based on in situ organothiol transformation in lithium sulfur battery. Nat Commun 2021; 12: 3031. 10.1038/s41467-021-23155-3 34050171 PMC8163853

[10] Chen H, Pei A, Lin D et al. Uniform high ionic conducting lithium sulfide protection layer for stable lithium metal anode. Adv Energy Mater 2019; 9: 1900858. 10.1002/aenm.201900858

[11] Chen W, Salvatierra R, Li J et al. Brushed metals for rechargeable metal batteries. Adv Mater 2022; 34: 2202668. 10.1002/adma.202202668 35709635

[12] Li Z, Zhang J, Lu Y et al. A pyrolyzed polyacrylonitrile/selenium disulfide composite cathode with remarkable lithium and sodium storage performances. Sci Adv 2018; 4: eaat1687. 10.1126/sciadv.aat1687 29888331 PMC5993473

[13] Chen X, Peng L, Wang L et al. Ether-compatible sulfurized polyacrylonitrile cathode with excellent performance enabled by fast kinetics via selenium doping. Nat Commun 2019; 10: 1021. 10.1038/s41467-019-08818-6 30833552 PMC6399341

[14] Wang W, Xi K, Li B et al. A sustainable multipurpose separator directed against the shuttle effect of polysulfides for high-performance lithium-sulfur batteries. Adv Energy Mater 2022; 12: 2200160. 10.1002/aenm.202200160

[15] Luo C, Zhu Y, Wen Y et al. Carbonized polyacrylonitrile-stabilized SeS_x_ cathodes for long cycle life and high power density lithium ion batteries. Adv Funct Mater 2014; 24: 4082–9. 10.1002/adfm.201303909

[16] Zhou J, Qian T, Xu N et al. Selenium-doped cathodes for lithium-organosulfur batteries with greatly improved volumetric capacity and coulombic efficiency. Adv Mater 2017; 29: 1701294. 10.1002/adma.201701294 28691212

[17] Tu Z, Choudhury S, Zachman M et al. Fast ion transport at solid-solid interfaces in hybrid battery anodes. Nat Energy 2018; 3: 310–6. 10.1038/s41560-018-0096-1

[18] Zhou Y, Zhang X, Ding Y et al. Redistributing Li-ion flux by parallelly aligned holey nanosheets for dendrite-free Li metal anodes. Adv Mater 2020; 32: 2003920. 10.1002/adma.202003920 32789959

[19] Lee D, Sun S, Kwon J et al. Copper nitride nanowires printed Li with stable cycling for Li metal batteries in carbonate electrolytes. Adv Mater 2020; 32: 1905573. 10.1002/adma.201905573 31930614

[20] Pathak R, Chen K, Gurung A et al. Fluorinated hybrid solid-electrolyte-interphase for dendrite-free lithium deposition. Nat Commun 2020; 11: 93. 10.1038/s41467-019-13774-2 31900398 PMC6941966

[21] Yin Y, Wang Q, Yang J et al. Metal chloride perovskite thin film based interfacial layer for shielding lithium metal from liquid electrolyte. Nat Commun 2020; 11: 1761. 10.1038/s41467-020-15643-9 32273513 PMC7145840

[22] Wang G, Chen C, Chen Y et al. Self-stabilized and strongly adhesive supramolecular polymer protective layer enables ultrahigh-rate and large-capacity lithium-metal anode. Angew Chem Int Ed 2020; 59: 2055–60. 10.1002/anie.201913351 31729145

[23] Zhang K, Liu W, Gao Y et al. A high-performance lithium metal battery with ion-selective nanofluidic transport in a conjugated microporous polymer protective layer. Adv Mater 2021; 33: 2006323. 10.1002/adma.202006323 33326157

[24] Wu J, Rao Z, Liu X et al. Polycationic polymer layer for air-stable and dendrite-free Li metal anodes in carbonate electrolytes. Adv Mater 2021; 33: 2007428. 10.1002/adma.202007428 33543568

[25] Gao R, Yang H, Wang C et al. Fatigue-resistant interfacial layer for safe lithium metal batteries. Angew Chem Int Ed 2021; 60: 25508–13. 10.1002/anie.202111199 34580988

[26] Chen C, Liang Q, Chen Z et al. Phenoxy radical-induced formation of dual-layered protection film for high-rate and dendrite-free lithium-metal anodes. Angew Chem Int Ed 2021; 60: 26718–24. 10.1002/anie.202110441 34580969

[27] Zhao Q, Chen X, Hou W et al. A facile, scalable, high stability lithium metal anode. Susmat 2022; 2: 104–12. 10.1002/sus2.43

[28] Guo F, Wu C, Chen H et al. Dendrite-free lithium deposition by coating a lithiophilic heterogeneous metal layer on lithium metal anode. Energy Storage Mater 2020; 24: 635–43. 10.1002/anie.202116586

[29] Jin X, Cai Z, Zhang X et al. Transferring liquid metal to form a hybrid solid electrolyte via a wettability-tuning technology for lithium-metal anodes. Adv Mater 2022; 34: 2200181. 10.1002/adma.202200181 35238080

[30] Li S, Huang J, Cui Y et al. A robust all-organic protective layer towards ultrahigh-rate and large-capacity Li metal anodes. Nat Nanotechnol 2022; 17: 613–21. 10.1038/s41565-022-01107-2 35469010

[31] Zhao F, Zhai P, Wei Y et al. Constructing artificial SEI layer on lithiophilic MXene surface for high-performance lithium metal anodes. Adv Sci 2022; 9: 2103930. 10.1002/advs.202103930 PMC8867166 34990077

[32] Chang S, Jin X, He Q et al. *In situ* formation of polycyclic aromatic hydrocarbons as an artificial hybrid layer for lithium metal anodes. Nano Lett 2022; 22: 263–70. 10.1021/acs.nanolett.1c03624 34905368

[33] Cui Y, Liu S, Wang D et al. A facile way to construct stable and ionic conductive lithium sulfide nanoparticles composed solid electrolyte interphase on Li metal anode. Adv Funct Mater 2021; 31: 2006380.

[34] Lu W, Sun L, Zhao Y et al. Elongating the cycle life of lithium metal batteries in carbonate electrolyte with gradient solid electrolyte interphase layer. Energy Storage Mater 2021; 34: 241–9. 10.1016/j.ensm.2020.09.017

[35] Liu P, Zhang J, Zhong L et al. Interphase building of organic-inorganic hybrid polymer solid electrolyte with uniform intermolecular Li^+^ path for stable lithium metal batteries. Small 2021; 17: 2102454. 10.1002/smll.202102454 34514698

[36] Lee D, Sun S, Park H et al. Stable artificial solid electrolyte interphase with lithium selenide and lithium chloride for dendrite-free lithium metal anodes. J Power Sources 2021; 506: 230158. 10.1016/j.jpowsour.2021.230158

[37] Efaw C, Lu B, Lin Y et al. A closed-host bi-layer dense/porous solid electrolyte interphase for enhanced lithium-metal anode stability. Mater Today 2021; 49: 48–58. 10.1016/j.mattod.2021.04.018

[38] Zhang Y, Sun C. Composite lithium protective layer formed *in situ* for stable lithium metal batteries. ACS Appl Mater Interfaces 2021; 13: 12099–105. 10.1021/acsami.1c00745 33653027

[39] Wen Z, Fang W, Wu X et al. High-concentration additive and triiodide/iodide redox couple stabilize lithium metal anode and rejuvenate the inactive lithium in carbonate-based electrolyte. Adv Funct Mater 2022; 32: 2204768. 10.1002/adfm.202204768

[40] Sheng L, Wang Q, Liu X et al. Suppressing electrolyte-lithium metal reactivity via Li^+^-desolvation in uniform nano-porous separator. Nat Commun 2022; 13: 172. 10.1038/s41467-021-27841-0 35013293 PMC8748786

[41] Chen M, Zheng J, Liu Y et al. Marrying ester group with lithium salt: cellulose-acetate-enabled LiF-enriched interface for stable lithium metal anodes. Adv Funct Mater 2021; 31: 2102228. 10.1002/adfm.202102228

[42] Xu Q, Yang X, Rao M et al. High energy density lithium metal batteries enabled by a porous graphene/MgF_2_ framework. Energy Storage Mater 2020; 26: 73–82. 10.1016/j.ensm.2019.12.028

[43] Liang J, Li X, Zhao Y et al. *In situ* Li_3_PS_4_ solid-state electrolyte protection layers for superior long-life and high-rate lithium-metal anodes. Adv Mater 2018; 30: 1804684. 10.1002/adma.201804684 30276879

[44] Liu F, Wang L, Zhang Z et al. A mixed lithium-ion conductive Li_2_S/Li_2_Se protection layer for stable lithium metal anode. Adv Funct Mater 2020; 30: 2001607. 10.1002/adfm.202001607

[45] Tan S, Jiang Y, Ni S et al. Serrated lithium fluoride nanofibers-woven interlayer enables uniform lithium deposition for lithium-metal batteries. Natl Sci Rev 2022; 9: nwac183. 10.1093/nsr/nwac183 36381218 PMC9647010

[46] Fang R, Xu B, Grundish N et al. Li_2_S_6_-integrated PEO-based polymer electrolytes for all-solid-state lithium-metal batteries. Angew Chem Int Ed 2021; 60: 17701–6. 10.1002/anie.202106039 34192402

