# Supplementary material for: A versatile reactive layer toward ultra-long lifespan lithium metal anodes
Source: Natl Sci Rev. 2024 Dec 3;12(2):nwae421. doi: 10.1093/nsr/nwae421 (PMC11737383; doi:10.1093/nsr/nwae421)
Supplement: nwae421_Supplemental_File [file nwae421_supplemental_file.pdf]

Supplementary Information

A versatile reactive layer toward ultra-long lifespan lithium metal anodes

*Jinlun Wu, Yuheng Lu, Xianlan Ke, Linying Zheng, Rongfeng Liao, and Dingcai Wu**

PCFM Lab and GDHPRC Lab, School of Chemistry, Sun Yat-sen University; Guangzhou, 510006, P. R. China

* Email: wudc@mail.sysu.edu.cn

These authors contributed equally: Jinlun Wu, Yuheng Lu, Xianlan Ke.

Methods

Materials. Carbon nanotube (CNT; Guangdong Canrd New Energy Technology, 7321), hydrochloric acid (Guangzhou Chemical Reagent, 36.0%-38.0%), para-phenylenediamine (Aladdin, 97%), sodium nitrite (Macklin, 99.99%), tetrahydrofuran (THF; Macklin, 99.5%), acryloyl chloride (Aladdin, 96%), acetone (Guangzhou Chemical Reagent, 99.5%), N, N-dimethylformamide (DMF; Aladdin, 99.5%), acrylonitrile (Macklin, 99%), azobisisobutyronitrile (AIBN; Aladdin, 99%), SeS_2 (Macklin, 97%), polyvinylidene difluoride (PVDF; Sigma-Aldrich, M_w ≈ 534000), and N-methylpyrrolidone (NMP; Aladdin, 99.9%).

Synthesis of CNT-*g*-PAN. 700 mg CNT was dispersed in 250 mL deionized water with addition of 10 mL hydrochloric acid. After ultra-sonication for 2 h, 1.733 g para-phenylenediamine was added and stirred for 0.5 h. Under ice bath, 444 mg sodium nitrite dissolved in 10 mL deionized water was added dropwise in 30 min, and the resulting mixture was further stirred at room temperature (25 ^oC) for 15 h. The as-obtained amino-modified CNT ($CNT-NH_2$) was vacuum filtrated and washed with deionized water and THF. 1.4 mL acryloyl chloride dissolved in 10 mL acetone was then added dropwise in the mixture of 350 mg $CNT-NH_2$ and 25 mL acetone for 30

min under ice bath. After stirring at room temperature (25 ^{o}C) for 24 h, CNT modified by vinyl group (CNT-C=C) was collected after vacuum filtration, followed by washing with deionized water and acetone. Thereafter, 350 mg CNT-C=C was dispersed in 40 mL DMF and sonicated for 30 min with addition of 12 mL purified acrylonitrile. After gentle N_2 purge for 30 min, 2.468 g AIBN was added and sealed in a 65 ^{o}C oil bath for 24 h. The polymerization reaction was stopped by opening the flask and exposing the catalyst to air. Finally, the as-obtained CNT-*g*-PAN was collected and washed with DMF to remove any free polyacrylonitrile polymers, followed by vacuum drying at 70 ^{o}C overnight.

Synthesis of CNT-*g*-*s*PAN. The mixture of CNT-*g*-PAN and commercial SeS_2 powder was put in an alumina crucible wrapped with aluminum foil. After heat treatment at 380 ^{o}C for 8 h under N_2 flow, the mixture in an opened alumina crucible was kept at 300 ^{o}C for another 4h to remove any SeS_2 surplus. Similarly, the CNT/*s*PAN control sample was prepared by heat treatment of the mixture of CNT, PAN and SeS_2 under identical fabrication conditions.

The weight ratio (X) of PAN in CNT-*g*-PAN or *s*PAN in CNT-*g*-*s*PAN was calculated according to the following equation:

$$X = \frac{C-B}{A-B} \times 100\%$$

where A, B and C represent the residual weight ratios of pure PAN/*s*PAN, CNT-C=C and CNT-*g*-PAN/CNT-*g*-*s*PAN after thermogravimetric analysis, respectively.

Preparation of CNT-*g*-*s*PAN@PP separator. Firstly, CNT-*g*-*s*PAN was mixed with PVDF binder with a mass ratio of 9:1 in NMP. After that, the slurry was doctor-bladed onto the one side of a commercial Celgard 2325 porous polyolefin (PP) separator. After vacuum drying at 70 ^{o}C for 12 h, CNT-*g*-*s*PAN@PP separator was obtained.

Material characterization. Field emission scanning electron microscopy (FESEM, Hitachi S-4800) and transmission electron microscopy (TEM, FEI Tecnai G2 F30) were used to characterize morphologies and structures of all samples. Elemental mappings were investigated by EDX spectroscopy attached to SEM and TEM instruments. TGA measurements were conducted under a N_2 atmosphere on a NETZSCH TG 209F1 Libra instrument (temperature range: 30-800 ^{o}C, heating rate:

10 ^{o}C min^{-1}). XRD patterns were measured by a D-MAX 2200 VPC diffractometer with Cu-Kα radiation at a scanning rate of 10 ° min^{-1} from 10 ° to 80 °. FT-IR spectra were recorded at room temperature on a Bruker Equinox 55. Raman spectra were examined by an inVia Qontor Raman spectroscope with a 532 nm excitation. TGA-mass spectroscopy (TG-MS) measurement was performed on a Thermo plus EV2/Thermo mass photo. In situ Raman measurement was performed in a Renishaw inVia Raman Spectrometer with an excitation wavelength of 532 nm by an Ar ion laser (50 mW). The microscope attachment was based on a Leica DMLM system using a 50 × objective; data acquisition and storage were carried out by a computer with Renishaw WiRE Raman software version 5.3. The time interval of curve collection was 397 s, and the Raman spectra were recorded in the range of 200-2000 cm^{-1}. XPS measurements were carried out on an ESCALAB Xi+ spectrometer (Thermo Fisher) using 250 W monochromatized Al Kα radiation. Contact angle measurements were performed by using a KRÜSS DSA100 instrument. TOF-SIMS characterization was conducted on a TOF SIMS 5-100 instrument (IONTOF Gmbh) after sampling procedures. The Ar-filled plastic bag was used to minimize air exposure during the sample transformations. A Cs^+ beam (1 keV, 60 nA) was used to sputter a 200 μm × 200 μm area for 900 s, and a Bi^{3+} beam (30 keV, 0.75 pA) was used to analyze a 50 μm × 50 μm area inside the Cs^+ sputtered area for depth profiling. The sputtering rate is ~1.2 nm s^{-1} on GaN.

Electrochemical measurements. All electrochemical measurements of CR2032 coin cells were performed at 30 ^{o}C. The electrolyte used for Li^+ conductivity (σ), Li^+ transference number (t_{Li+}) and Li deposition was 1.0 M lithium bis(trifluoromethylsulfonyl)imide (LiTFSI) in a mixture of 1,3-dioxalane (DOL) and dimethyl ether (DME) (1:1 by volume) with 1 wt% $LiNO_3$ additive. Li^+ conductivity was measured in the frequency range of 1 MHz to 0.1 Hz by AC impedance spectroscopy in an electrochemical station (CHI660E, Chenhua) with a stainless steel|separator|stainless steel cell configuration.

The Li^+ conductivity (σ) was calculated according to the following equation:

$$\sigma = \frac{L}{SR}$$

where L, S and R represent separator thickness, area of the stainless-steel platelet and bulk resistance, respectively.

The t_{Li+} was measured by chronoamperometry with a polarization voltage of 10 mV applied for 2000 s and calculated according to the following equation:

$$t_{Li^+} = \frac{I_S(\Delta V - I_0 R_0)}{I_0(\Delta V - I_S R_S)}$$

where ΔV is the polarization voltage, I_0 and I_S are the initial and steady-state currents, respectively, and R_0 and R_S are the initial and steady-state interfacial resistances obtained from electrochemical impedance spectroscopy (EIS), respectively.

The average coulombic efficiency (CE) was investigated in Li|Cu half cells using standard Aurbach protocol. The current density in all steps was 1 mA cm^{-2}. In detail, Li|Cu half cell was preconditioned with a 20 mAh cm^{-2} Li deposition/stripping cycle. Subsequently, 20 mAh cm^{-2} Li (Q_t) was plated on Cu substrate, followed by repeated stripping/platting of l mAh cm^{-2} Li (Q_c) for 200 cycles. Finally, the residual Li (Q_s) was stripped out until 1 V. The CE was calculated according to the following equation:

$$CE = \frac{200Q_c + Q_s}{200Q_c + Q_t}$$

Li|Li symmetric cells were prepared with two Li foils (thickness = 450 μm) as working and counter electrodes. Cyclic voltammetry (CV) and linear sweep voltammetry (LSV) were conducted at a scan rate of 0.2 and 5 mV s^{-1}, respectively, using a CHI660E electrochemical workstation. As for Li-S cells, cathode material (BP2000/S) was prepared by mixing BP2000 (Guangzhou Lige Technology) and commercial sulfur (Aladdin, 99.0%) with a mass ratio of 1:4 with the heat treatment at 155 ℃ for 12 h. Sulfur cathode was prepared by mixing 70 wt% BP2000/S, 10 wt% super P (Guangdong Canrd New Energy Technology), 10 wt% EPC-600JD (Guangdong Canrd New Energy Technology) and 10 wt% PVDF in NMP. The slurry was dropped onto commercial carbon paper, followed by drying at 60 ℃ for 12 h. Unless otherwise specified, the sulfur loading of cathodes was controlled at 1 mg cm^{-2}. For regular cells, 80 μL electrolyte was applied. The electrolyte was 1.0 M LiTFSI in

a mixture of DOL and DME with 1 wt% $LiNO_3$ additive (Suzhou Duoduo Chemical Technology, LS-002). For Li|NCM622 cells, the loading of the NCM622 cathode was 21.6 mg cm^{-2} and the electrolyte was 40 μL of 1.0 M $LiPF_6$ in ethylene carbonate/diethyl carbonate/ethyl methyl carbonate (Guangdong Canrd New Energy Technology, KLD-1230C). For Li|NCM622 pouch cell, the sizes of Li foil (50 μm) and cathode (31.5 mg cm^{-2}) were 3.8 × 5.8 and 3.6 × 5.6 cm^2, respectively. The electrolyte used for pouch cell was consistent with the coin cell, and the amount was 1.2 g. The voltage windows for the Li-S and Li|NCM622 cells were set to 1.7-2.8 and 3.0-4.3 V, respectively. CV curves of Li-S cells were recorded at a scan rate of 0.1 mV s^{-1} in a voltage window of 1.7-2.8 V at an electrochemical station. EIS measurements were performed by using a CHI660E electrochemistry workstation with an AC amplitude of 5 mV over a frequency range of 100 kHz to 0.01 Hz.

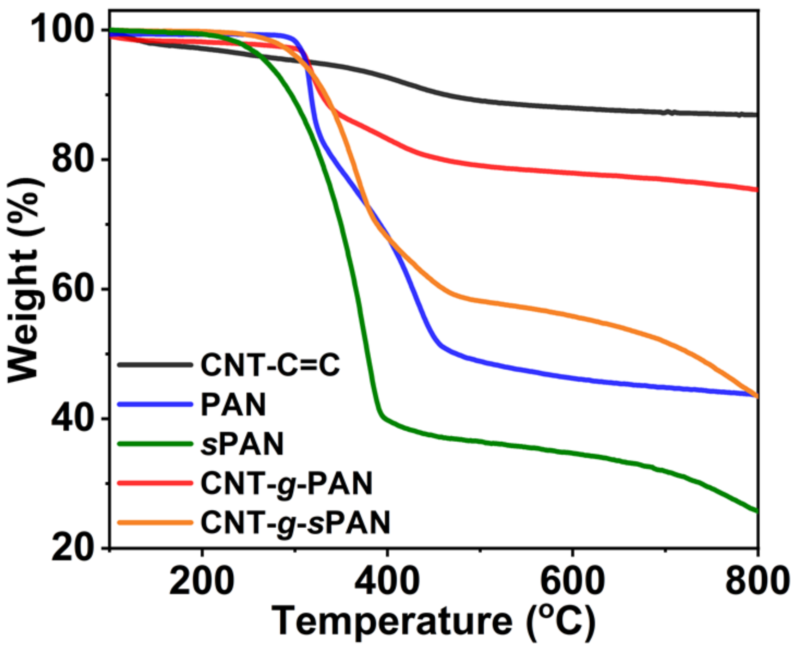

Supplementary Fig. 1. TGA curves of CNT-C=C, PAN, *s*PAN, CNT-*g*-PAN and CNT-*g*-*s*PAN.

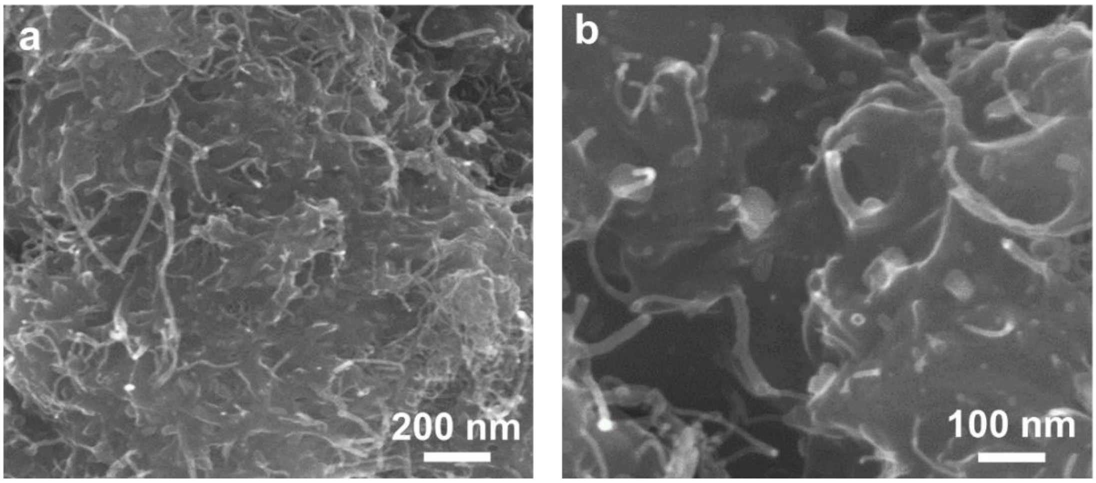

Supplementary Fig. 2. SEM images of CNT/*s*PAN.

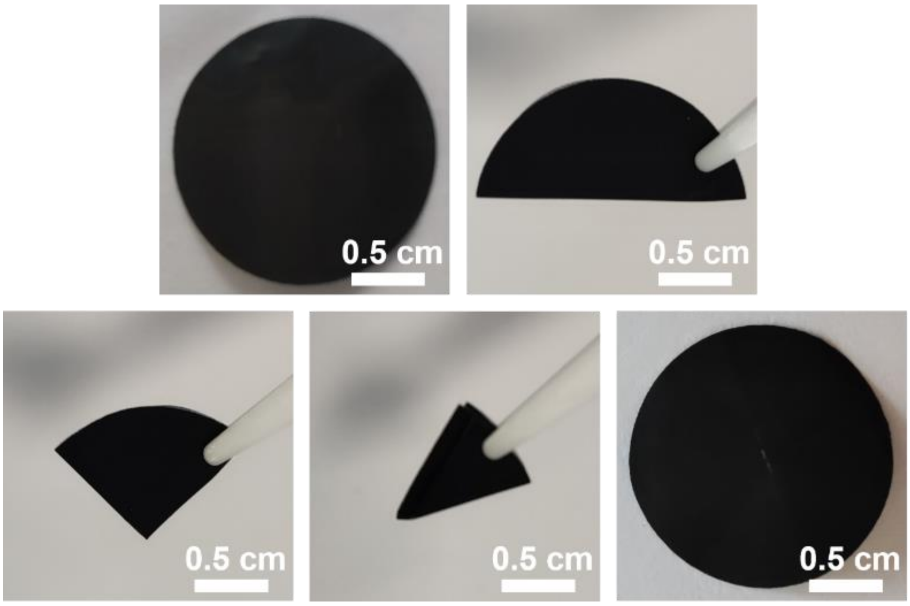

Supplementary Fig. 3. Digital photos of the CNT-*g*-*s*PAN@PP separator in different bending states.

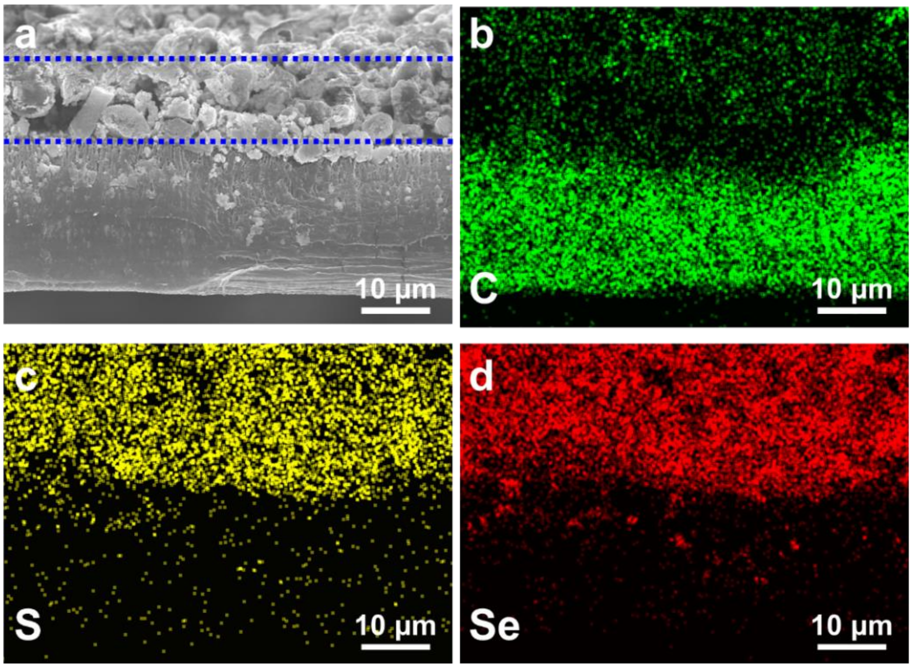

Supplementary Fig. 4. (a) Cross-section SEM image and (b-d) corresponding elemental mapping analysis of CNT-*g*-*s*PAN@PP separator.

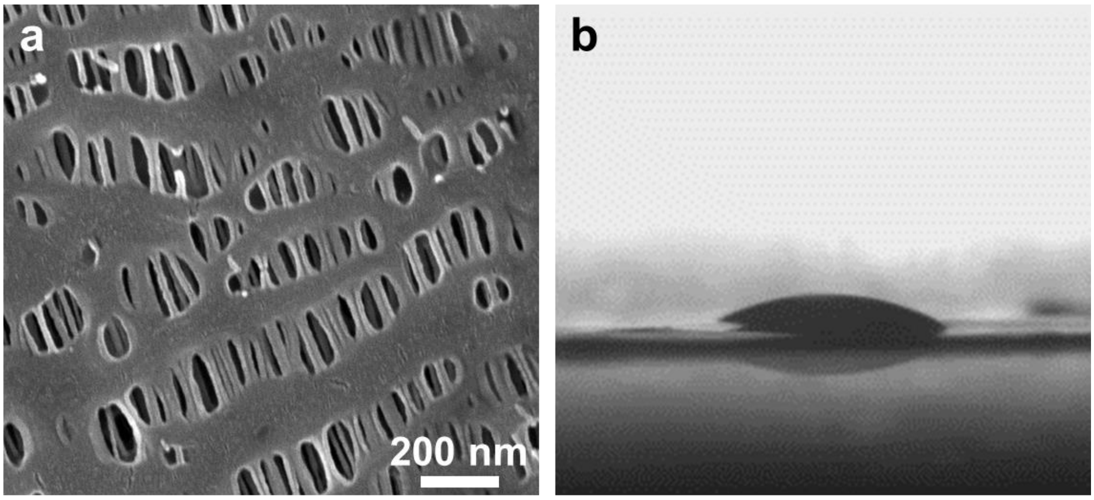

Supplementary Fig. 5. (a) Top-view SEM image and (b) electrolyte contact angle of PP separator.

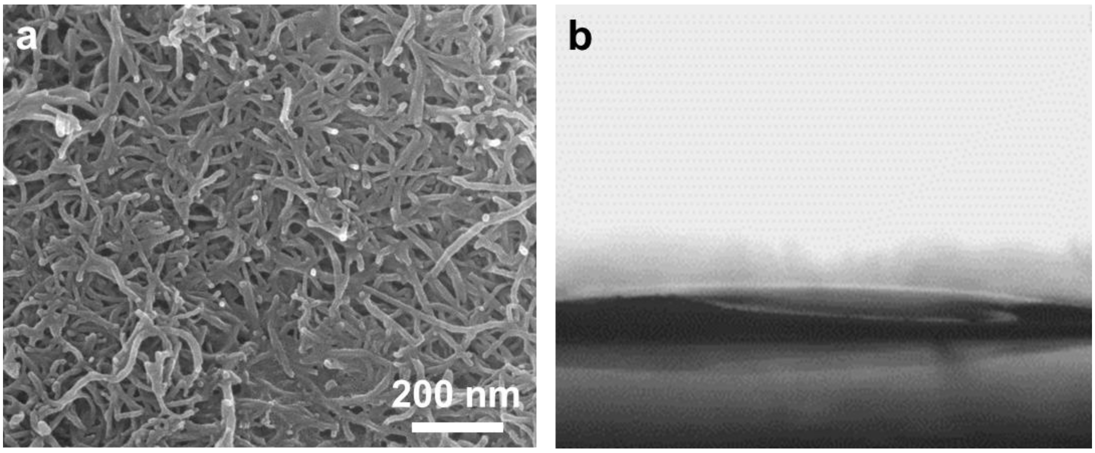

Supplementary Fig. 6. (a) Top-view SEM image and (b) electrolyte contact angle of CNT-*g*-*s*PAN@PP separator.

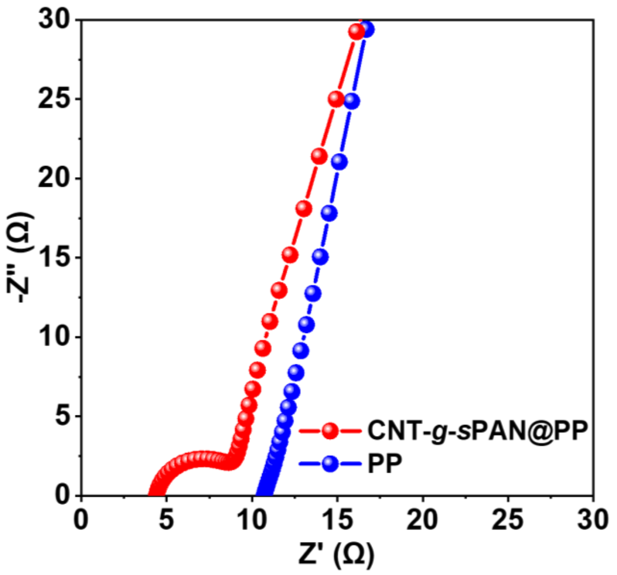

Supplementary Fig. 7. Li ion conductivity curves of CNT-*g*-*s*PAN@PP and PP separators.

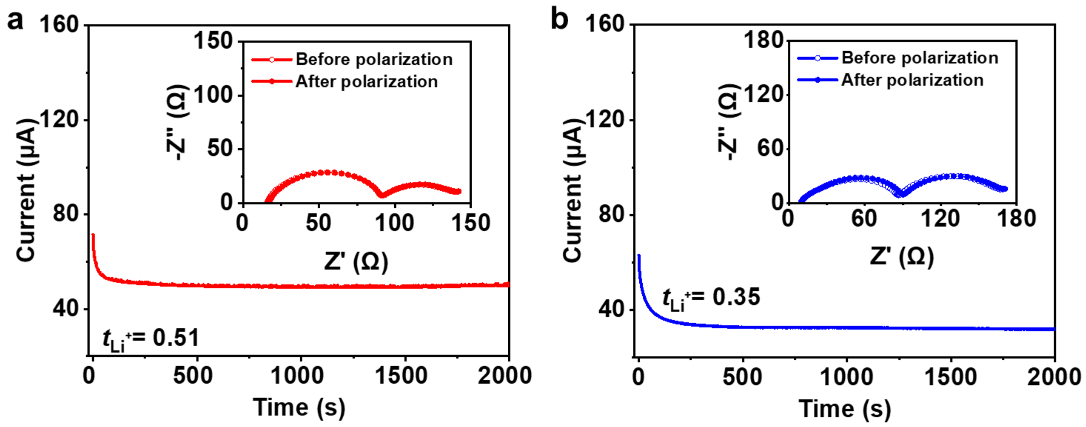

Supplementary Fig. 8. Chronoamperometry profiles of symmetric cells with (a) CNT-*g*-*s*PAN@PP and (b) PP under a polarization voltage of 10 mV. The inset shows their corresponding Nyquist plots before and after polarization.

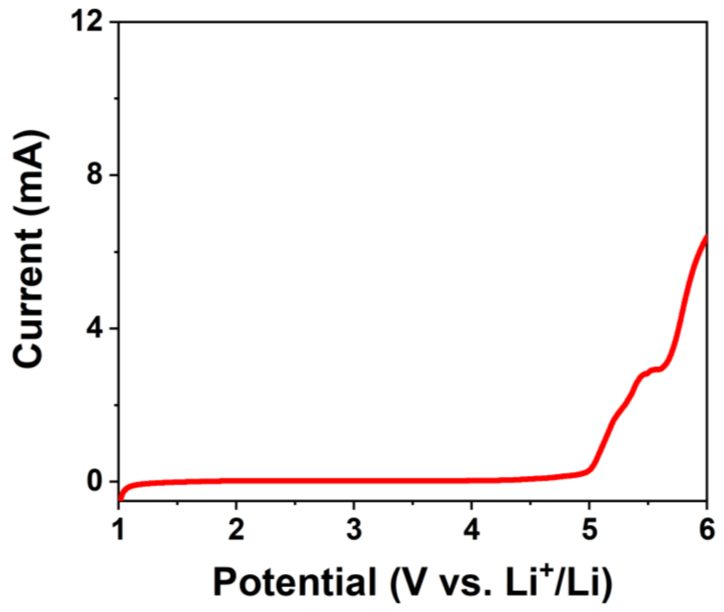

Supplementary Fig. 9. LSV curve of Li|stainless-steel cell with CNT-*g*-*s*PAN@PP separator at a scan rate of 5 mV s^{-1}.

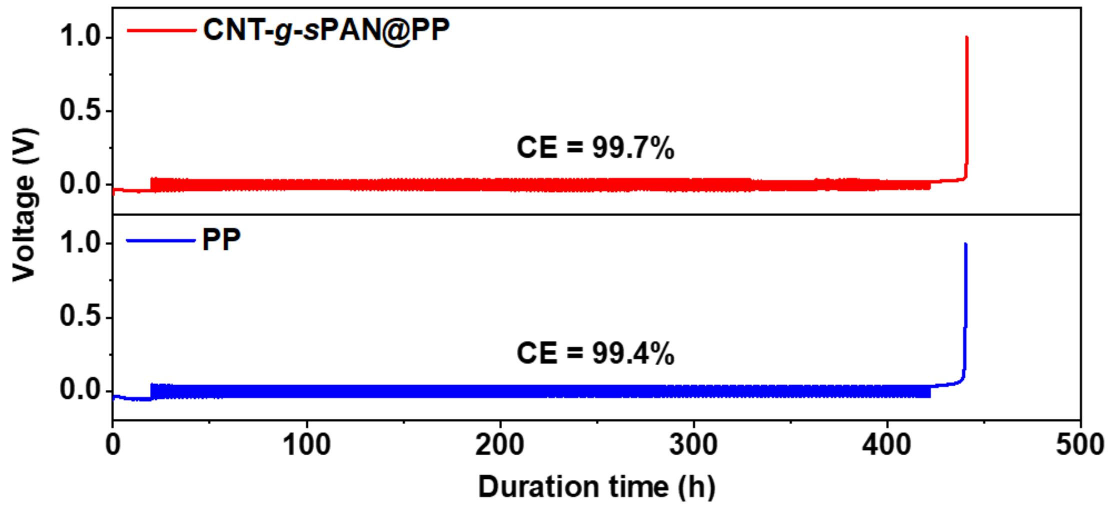

Supplementary Fig. 10. Coulombic efficiencies of Li|Cu cells with PP and CNT-*g*-*s*PAN@PP separators at 1 mAh cm^{-2} and 1 mA cm^{-2}.

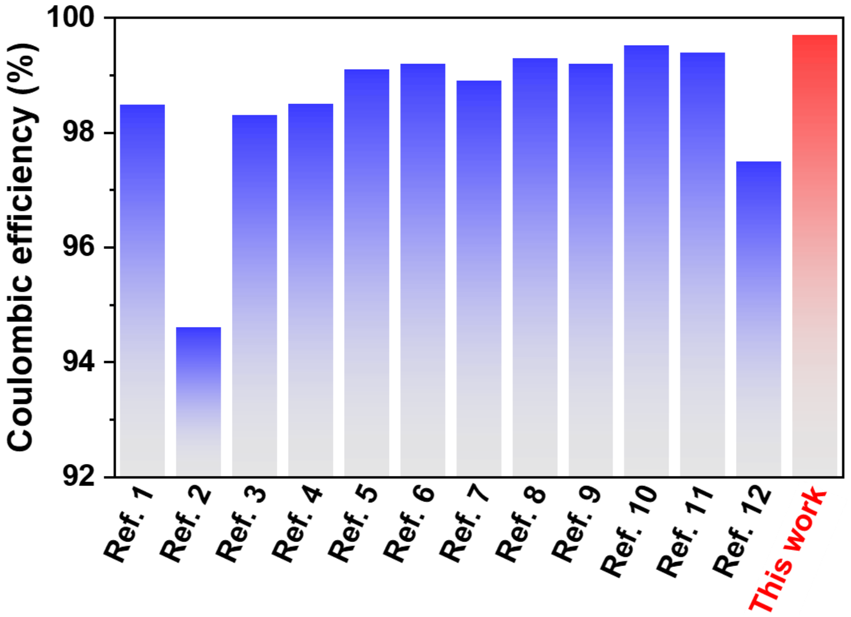

Supplementary Fig. 11. Average Coulombic efficiency of Li|Cu cell with CNT-*g*-*s*PAN separator, which is favorably comparable to those of reported asymmetric Li|Cu cells.

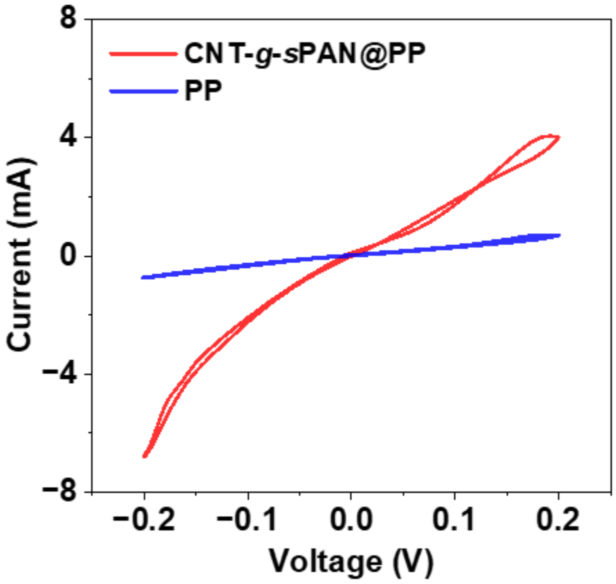

Supplementary Fig. 12. CV curves of Li|Li symmetrical cells with CNT-*g*-*s*PAN@PP and PP separators.

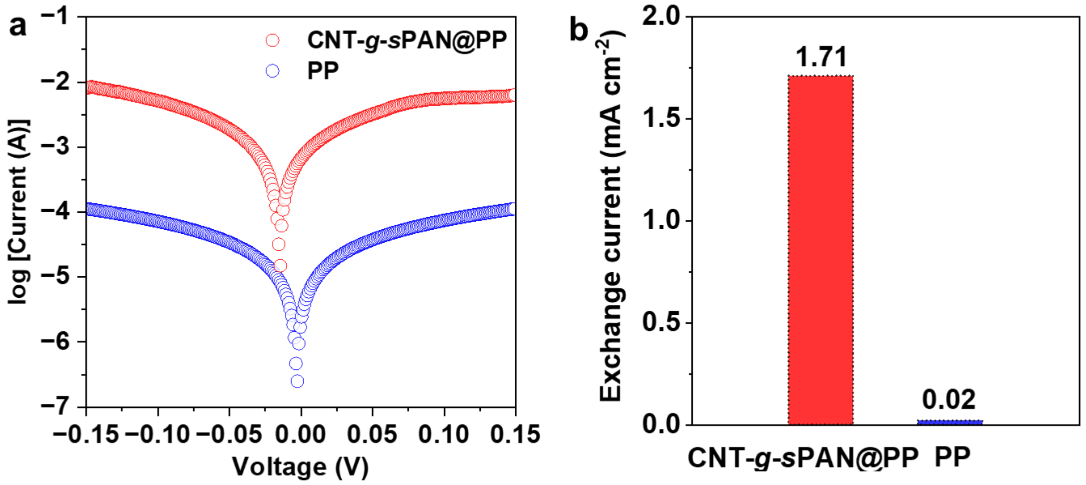

Supplementary Fig. 13. (a) Tafel curves and (b) corresponding exchange current densities of Li|Li symmetrical cells with CNT-*g*-*s*PAN@PP and PP separators.

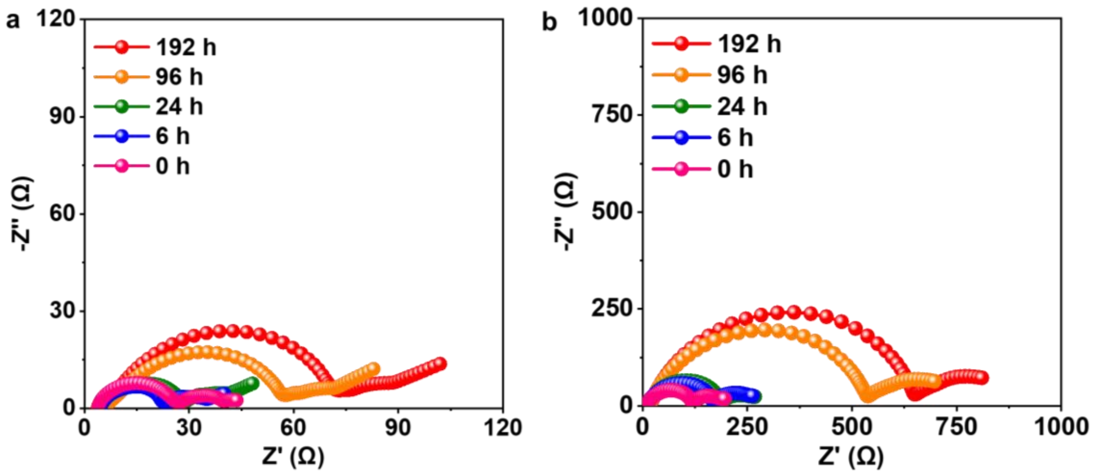

Supplementary Fig. 14. Nyquist plots as a function of standing time for Li|Li symmetric cells with (a) CNT-*g*-*s*PAN@PP and (b) PP separators.

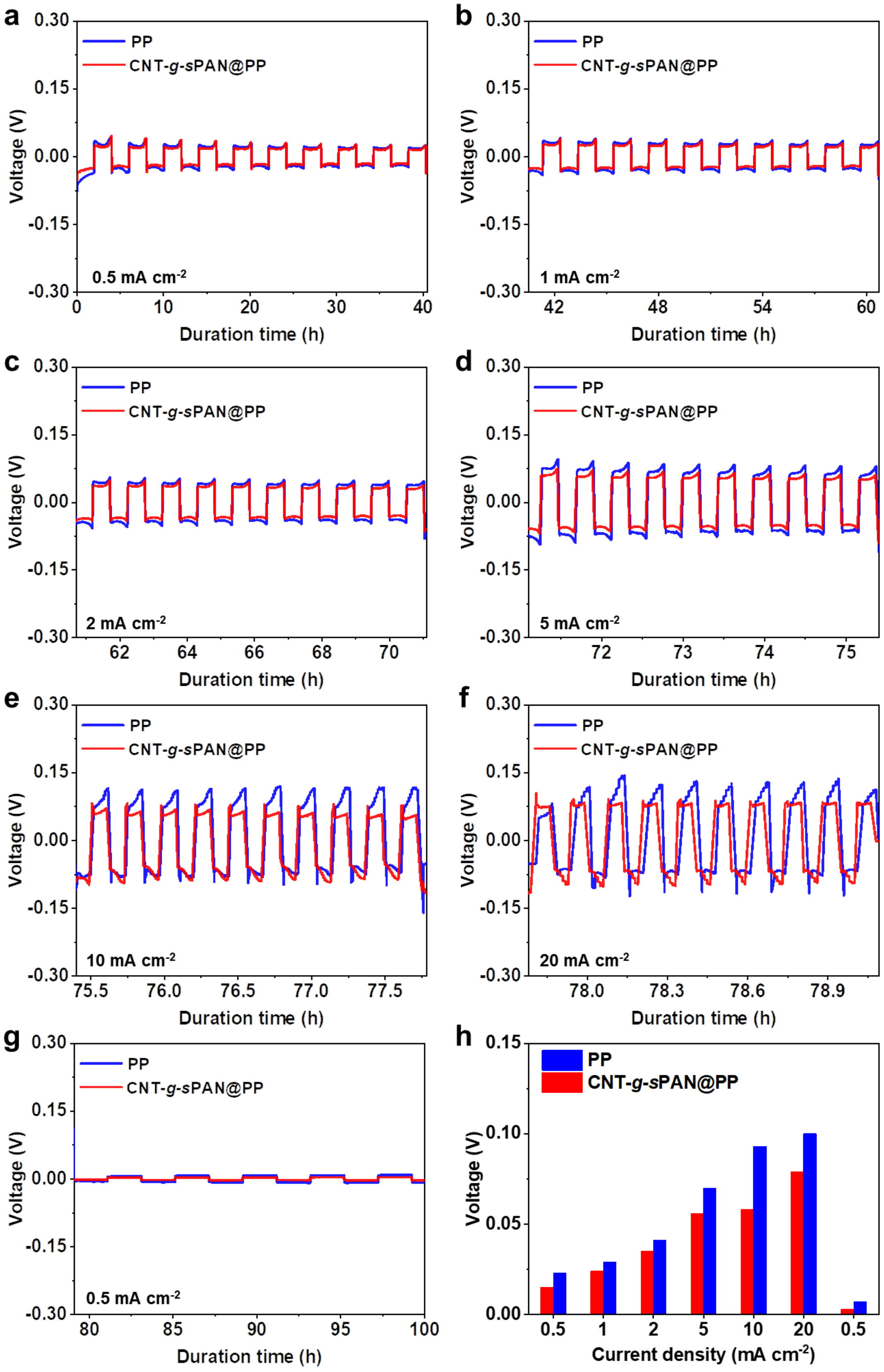

Supplementary Fig. 15. (a-g) Enlarged views in voltage-time profiles and (h) the voltages of Li/Li symmetric cells with CNT-*g*-*s*PAN@PP and PP separators at various current densities in a capacity of 1 mAh cm^{-2}.

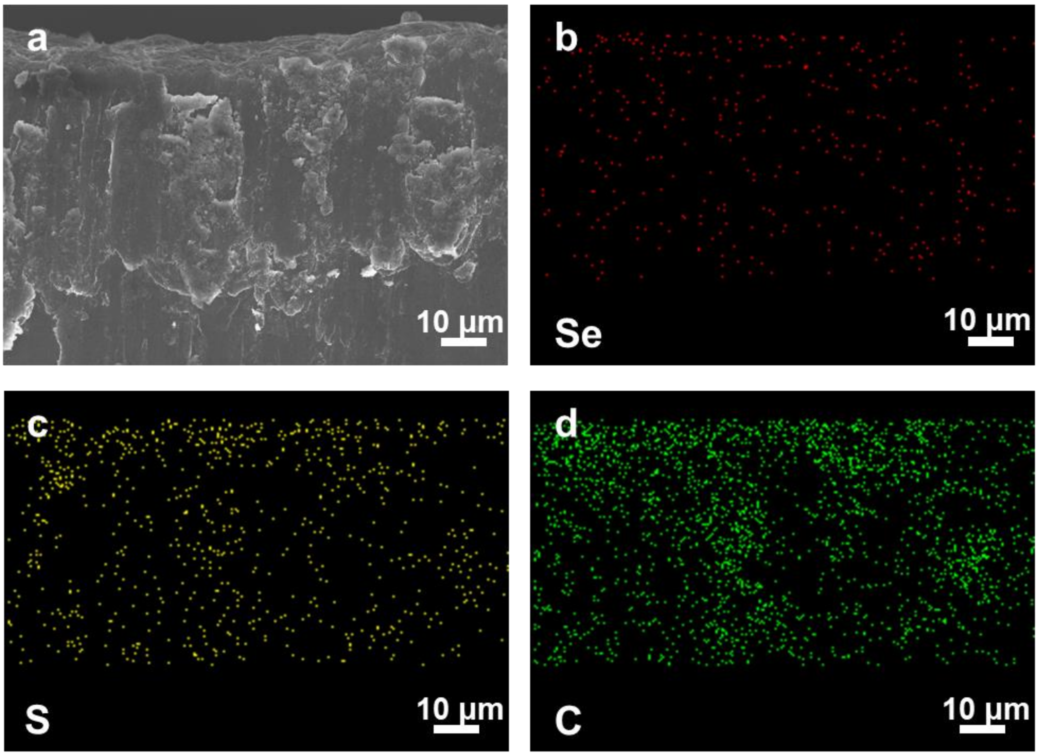

Supplementary Fig. 16. (a) Cross-section SEM image and (b-d) corresponding elemental mapping analysis of Li anode after the cycling test with CNT-*g*-*s*PAN@PP at 1 mAh cm^{-2} and 10 mA cm^{-2} for 100 cycles.

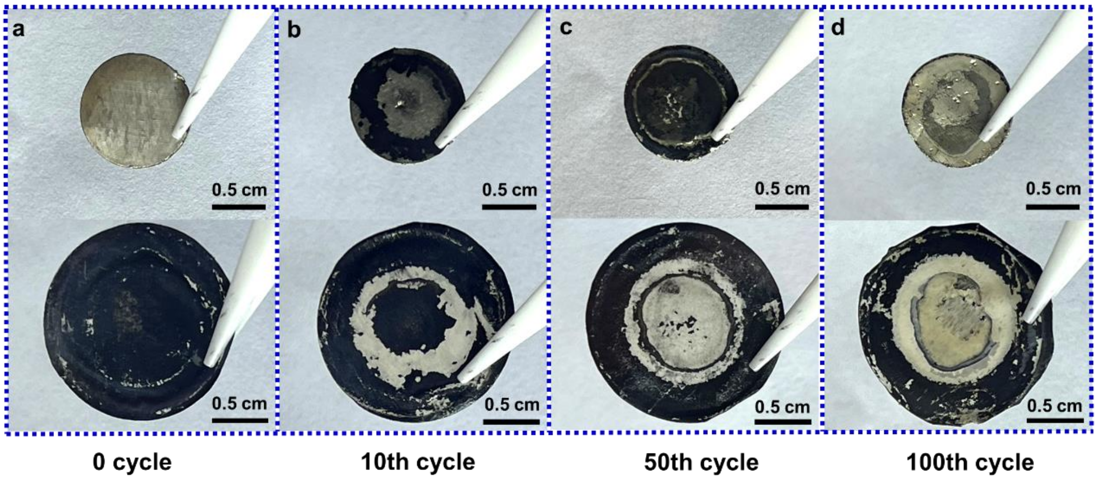

Supplementary Fig. 17. Digital images of Li anode and CNT-*g*-*s*PAN@PP separator in Li/Li symmetric cell after (a) 0, (b) 10, (c) 50, and (d) 100 cycles at 1 mAh cm^{-2} and 10 mA cm^{-2}.

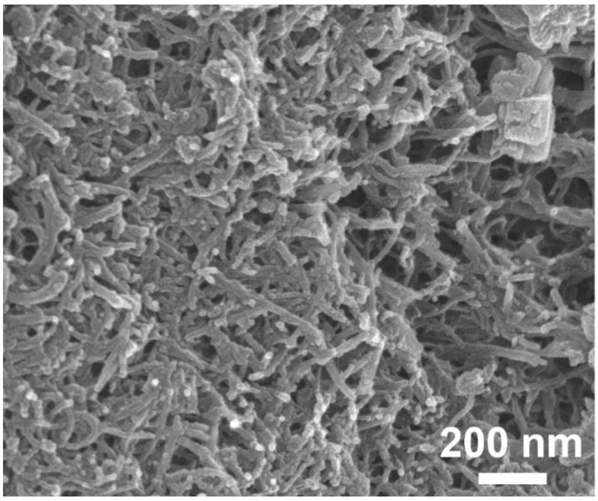

Supplementary Fig. 18. SEM image of the corresponding residuals derived from Li anode after 100 cycles at 1 mAh cm^{-2} and 10 mA cm^{-2}.

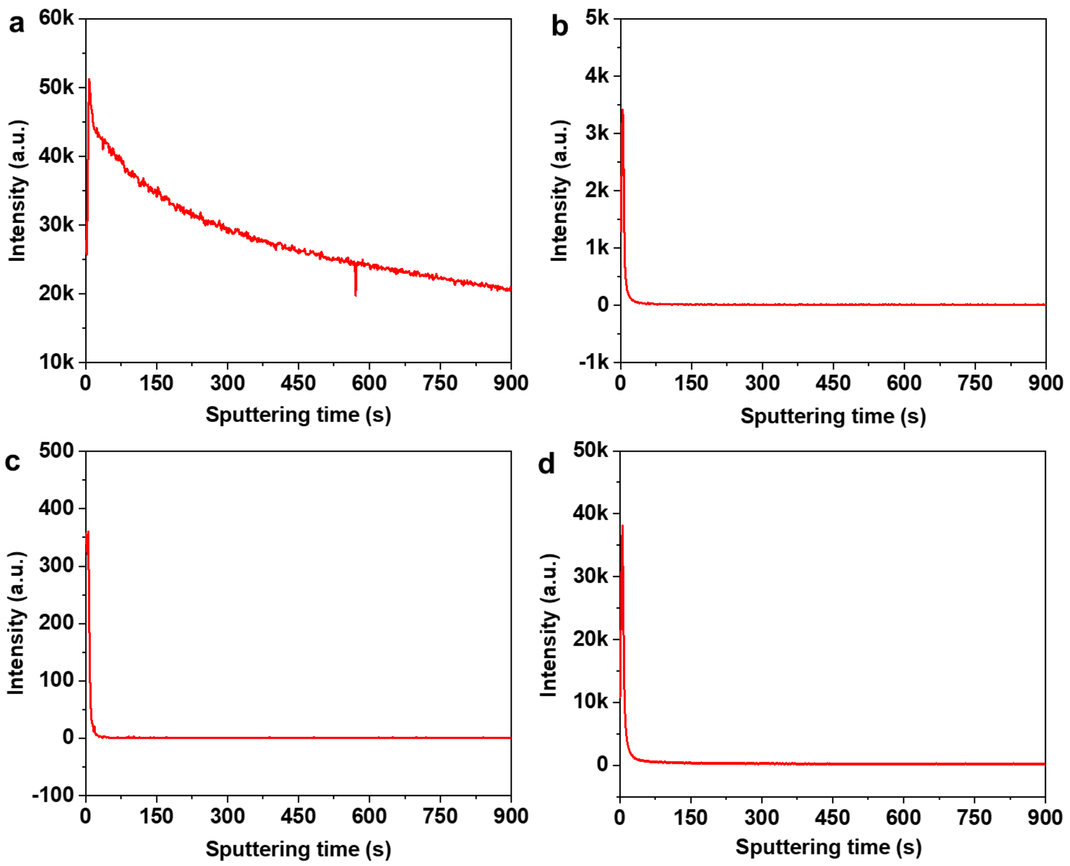

Supplementary Fig. 19. Depth profiles for (a) CN^-, (b) Li_2S^-, (c) Li_2Se^- and (d) LiF_2^- secondary ions.

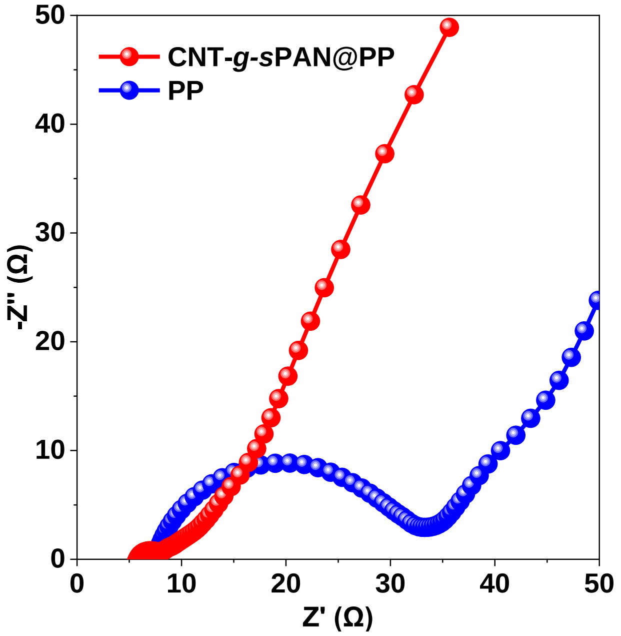

Supplementary Fig. 20. Nyquist plots of Li-S cells with PP and CNT-*g*-sPAN@PP separators.

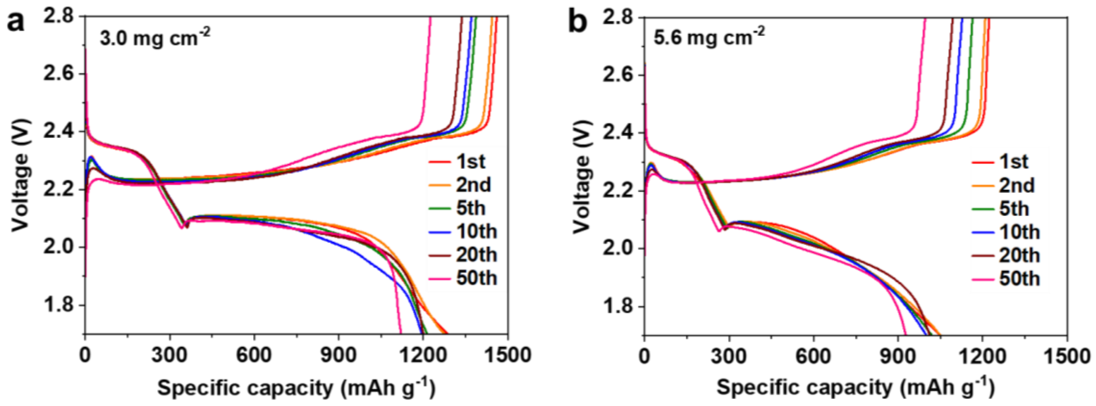

Supplementary Fig. 21. Galvanostatic charge-discharge profiles of Li-S cell with CNT-*g*-sPAN@PP separator in various cycles at 0.1 C under elevated sulfur loadings: (a) 3.0 and (b) 5.6 mg cm^{-2}.

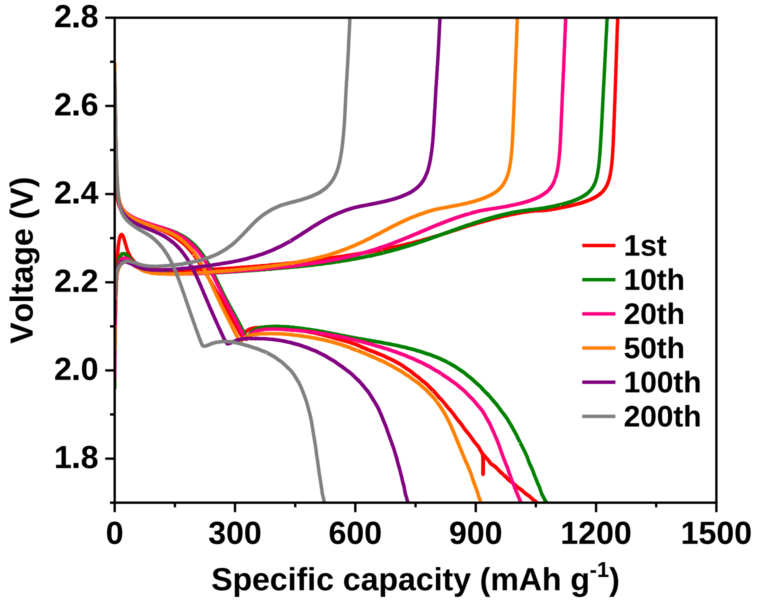

Supplementary Fig. 22. Galvanostatic charge-discharge profiles of CNT-*g*-sPAN@PP separator-based Li-S cell with an ultrathin Li anode (50 μm) and a high sulfur loading cathode (4.5 mg cm^{-2}) in various cycles at 0.1 C.

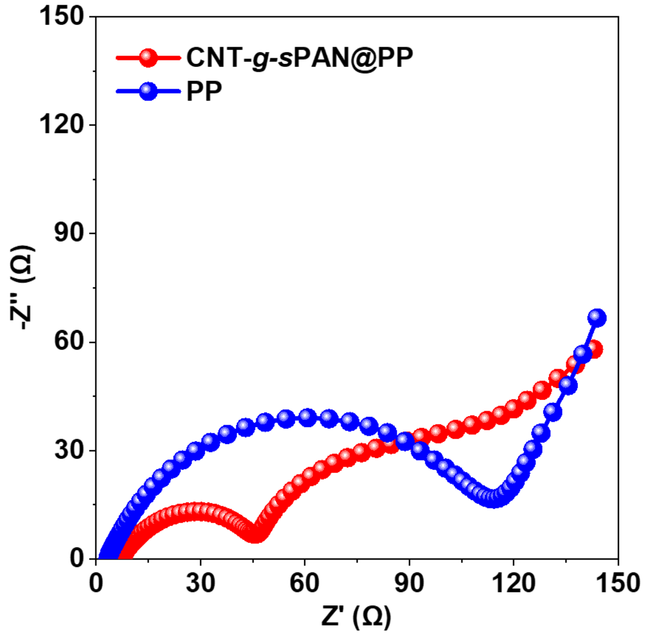

Supplementary Fig. 23. Nyquist plots of Li|NCM622 cells with PP and CNT-*g*-sPAN@PP separators.

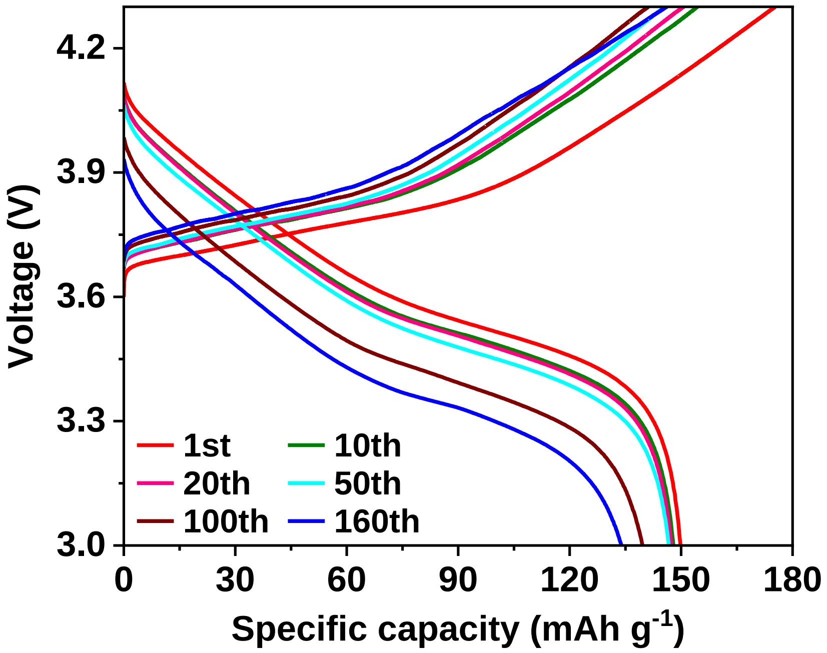

Supplementary Fig. 24. Galvanostatic charge-discharge (0.2/1 C) profiles of Li|NCM622 cell with CNT-*g*-*s*PAN@PP separator under a high loading of 21.6 mg cm^{-2}.

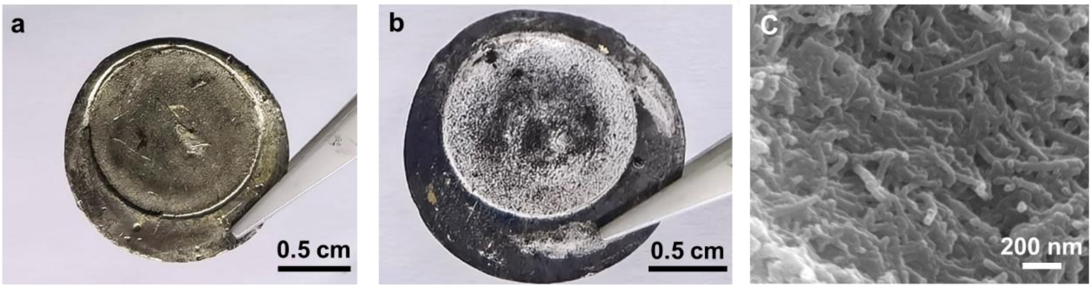

Supplementary Fig. 25. Digital images of (a) Li anode and (b) CNT-*g*-*s*PAN@PP separator after cycling for 100 cycles in Li|NCM622 full cell at 1 C. (c) SEM image of the corresponding residuals derived from the cycled Li anode.

Supplementary Table 1. Comparison of electrochemical performances between our Li-S cells and reported Li-S cells.

Samples	Li (μm)	S loading (mg cm^{-2})	N/P ratio	Capacity (mAh g^{-1})	Rate (C)	Cycle number	Refs.
CNT-*g*-*s*PAN	50	4.5	1.4	1033	0.1	200	This work
HFE-EPSE	50	4.4	1.4	<957	0.1	135	13
1,4-BDT	--	8.7	--	830	0.1	200	14
PDMS-Li	--	2.5		1139	0.6	100	15
r-GO/Li	50	1.0	6.0	770	0.6	100	16
ISDN	50	4.0	1.5	892	0.1	100	17
EPSE	50	4.0	1.5	889 (5th)	0.1	120	18
DPDSe	33	1.2	4.5	1056	0.5	350	19
D-Cu@CuSe	--	4	2	<1000	0.5	30	20

References

1. L. Lin, *et al.* A better choice to achieve high volumetric energy density: anode-free lithium-metal batteries. *Adv. Mater.* **34**, 2110323 (2022).
2. C. Zhang, *et al.* An ultralight, pulverization-free integrated anode toward lithium-less lithium metal batteries. *Sci. Adv.* **10**, l4842 (2024).
3. X. Liu, *et al.* Locally concentrated ionic liquid electrolytes enabling low-temperature lithium metal batteries. *Angew. Chem. Int. Ed.* **62**, 202305840 (2023).
4. C. Chen, *et al.* Dynamic gel as artificial interphase layer for ultrahigh-rate and large-capacity lithium metal anode. *Nat. Commun.* **14**, 4018 (2023).
5. Y. Ma *et al.* A “blockchain” synergy in conductive polymer-filled metal–organic frameworks for dendrite-free Li plating/stripping with high Coulombic efficiency. *Angew. Chem. Int. Ed.* **61**, 202116291 (2022).
6. S. Li, *et al.* A robust all-organic protective layer towards ultrahigh-rate and large-capacity Li metal anodes. *Nat. Nanotechnol.* **17**, 613 (2022).
7. H. Liu, *et al*. In-situ constructing a heterogeneous layer on lithium metal anodes for dendrite-free lithium deposition and high Li-ion

flux. *Angew. Chem. Int. Ed.* **62**, 202217458 (2023).

8. L. Sheng, *et al.* Suppressing electrolyte-lithium metal reactivity via Li^+-desolvation in uniform nano-porous separator. *Nat. Commun.* **13**, 172 (2022).
9. J. Cao, *et al.* Hierarchical Li electrochemistry using alloytype anode for high-energy-density Li metal batteries. *Nat. Commun.* **15**, 1354 (2024).
10. Z. Yu, *et al.* Molecular design for electrolyte solvents enabling energy-dense and long-cycling lithium metal batteries. *Nat. Energy* **5**, 526 (2020).
11. J. Ding, *et al.* Non-solvating and low-dielectricity cosolvent for anion-derived solid electrolyte interphases in lithium metal batteries. *Angew. Chem. Int. Ed.* **60**, 11442 (2021).
12. S. Zhang, *et al.* A novel potassium salt regulated solvation chemistry Enabling excellent Li-anode protection in carbonate electrolytes. *Adv. Mater.* **35**, 2301312 (2023).
13. L. Hou, *et al.* Weakening the solvating power of solvents to encapsulate lithium polysulfides enables long-cycling lithium-sulfur batteries. *Adv. Mater.* **34**, 2205284 (2022).
14. J. Lian, *et al.* Isomeric organodithiol additives for improving interfacial chemistry in rechargeable Li-S batteries. *J. Am. Chem. Soc.* **143**, 11063 (2021).

15. Q. Li, *et al.* Poly(dimethylsiloxane) modified lithium anode for enhanced performance of lithium-sulfur batteries. *Energy Storage Mater.* **13**, 151 (2018).
16. A. Wang, *et al.* Bending-tolerant anodes for lithium-metal batteries. *Adv. Mater.* **30**, 1703891 (2018).
17. L. Hou, *et al.* Modification of nitrate ion enables stable solid electrolyte interphase in lithium metal batteries. *Angew. Chem. Int. Ed.* **61**, 202201406 (2022).
18. X. Zhang, *et al.* Electrolyte structure of lithium polysulfides with anti-reductive solvent shells for practical lithium-sulfur batteries. *Angew. Chem. Int. Ed.* **60**, 15503 (2021).
19. M. Zhao, *et al*. An organodiselenide comediator to facilitate sulfur redox kinetics in lithium-sulfur batteries. *Adv. Mater.* **33**, 2007298 (2021).
20. Z. Shi, *et al.* Synergizing conformal lithiophilic granule and dealloyed porous skeleton toward Pragmatic Li metal anodes. *Small Sci.* **2**, 2100110 (2022).
